# β-Catenin promotes long-term survival and angiogenesis of peripheral blood mesenchymal stem cells via the Oct4 signaling pathway

**DOI:** 10.1038/s12276-022-00839-4

**Published:** 2022-09-01

**Authors:** Pengzhen Wang, Zhanyu Deng, Aiguo Li, Rongsen Li, Weiguang Huang, Jin Cui, Songsheng Chen, Biao Li, Shaoheng Zhang

**Affiliations:** 1 Department of Cardiology, Guangzhou Red Cross Hospital, Jinan University, Guangzhou, Guangdong 510220 P.R. China; 2 Guangzhou Institute of Traumatic Surgery, Guangzhou Red Cross Hospital, Jinan University, Guangzhou, Guangdong 510220 P.R. China; 3 Department of Orthopaedics, Guangzhou Red Cross Hospital, Jinan University, Guangzhou, Guangdong 510220 P.R. China

**Keywords:** Myocardial infarction, Apoptosis, Mesenchymal stem cells, TOR signalling, Heart failure

## Abstract

Stem cell therapy has been extensively studied to improve heart function following myocardial infarction; however, its therapeutic potency is limited by low rates of engraftment, survival, and differentiation. Here, we aimed to determine the roles of the β-catenin/Oct4 signaling axis in the regulation of long-term survival and angiogenesis of peripheral blood mesenchymal stem cells (PBMSCs). These cells were obtained from rat abdominal aortic blood. We showed that β-catenin promotes the self-renewal, antiapoptotic effects, and long-term survival of PBMSCs by activating the Oct4 pathway through upregulation of the expression of the antiapoptotic factors Bcl2 and survivin and the proangiogenic cytokine bFGF and suppression of the levels of the proapoptotic factors Bax and cleaved caspase-3. β-Catenin overexpression increased Oct4 expression. β-Catenin knockdown suppressed Oct4 expression in PBMSCs. However, β-catenin levels were not affected by Oct4 overexpression or knockdown. Chromatin immunoprecipitation assays proved that β-catenin directly regulates Oct4 transcription in PBMSCs. In vivo, PBMSCs overexpressing β-catenin showed high survival in infarcted hearts and resulted in better myocardial repair. Further functional analysis identified Oct4 as the direct upstream regulator of Ang1, bFGF, HGF, VEGF, Bcl2, and survivin, which cooperatively drive antiapoptosis and angiogenesis of engrafted PBMSCs. These findings revealed the regulation of β-catenin in PBMSCs by the Oct4-mediated antiapoptotic/proangiogenic signaling axis and provide a breakthrough point for improving the long-term survival and therapeutic effects of PBMSCs.

## Heart disease: Extending the benefits of stem cell repair

Boosting expression of a specific gene has allowed researchers to generate stem cells with increased capacity for tissue repair after a heart attack. Several studies have shown that treatment with a population of circulating cells known as ‘peripheral blood mesenchymal stem cells’ (PBMSCs) can regenerate cardiac tissue. These cells generally have a short lifespan when used therapeutically, but researchers led by Shaoheng Zhang at Jinan University in Guangzhou China have increased long-term survival and performance by boosting expression of the gene encoding β-catenin, a protein that promotes cell survival and proliferation. PBMSCs expressing increased levels of β-catenin preserved heart function in a rat model of heart attack, stimulating blood vessel growth and improving animal survival. This study also reveals proteins regulated by β-catenin, which could potentially be exploited for finer control of PBMSC function.

## Introduction

Coronary heart disease is one of the deadliest and most prevalent diseases worldwide. Acute myocardial infarction (MI) is commonly encountered in hospitals and has high short-term and long-term morbidity and mortality rates. Mesenchymal stem cells (MSCs) are a promising cell type being evaluated for patients with MI. Peripheral blood MSCs (PBMSCs) have biological functions similar to those of bone marrow MSCs. However, the therapeutic potency of transplanted MSCs is limited by low rates of engraftment, survival, and differentiation^1^. Determining the mechanisms sustaining long-term proliferation and survival of MSCs is fundamental to developing new therapeutic treatments for MI.

β-Catenin, an important factor in leukemia stem cell activation and glioma progression^2^, is related to the transcriptional coactivation of molecules in the Wnt signaling pathway^3^. β-Catenin is vital for cancer stem cell (CSC) maintenance and self-renewal^4,5^. Inhibition of Wnt/β-catenin decreased the proliferation, survival, and clonogenic activity of T-cell acute lymphoblastic leukemia cells^6^. However, given the effects of β-catenin on CSCs, the precise role of Wnt/β-catenin signaling in MSC regulation remains unclear.

Wnt/β-catenin signaling might affect CSC biology by modulating the expression and activity of pluripotency and self-renewal-related transcription factors, namely, octamer-binding protein 4 (Oct4), Nanog, SRY box 2 (Sox2), and c-Myc^7,8^. Oct4, a POU isotopic transcription factor, is commonly expressed and activated in CSCs^9^. As an important protective factor for survival, Oct4 promotes MSC proliferation and inhibits cell apoptosis^10^. Previously, we suggested that Oct4 promotes the release of cytoprotective factors, such as basic fibroblast growth factor (bFGF), survivin, and Bcl2 (B-cell lymphoma-2). Therefore, Oct4 signaling might be the main contributor to maintaining the survival and function of MSCs^11^. However, whether Oct4-promoted MSC growth and antiapoptotic effects are regulated by β-catenin remains to be confirmed.

Based on the above considerations, we aimed to compare PBMSCs with β-catenin/Oct4 overexpression or knockdown. The protective effects of these PBMSCs in animal models of MI were also examined. Furthermore, we assessed the antiapoptotic and proangiogenic effects of β-catenin on PBMSCs by activating the Oct4 pathway.

## Materials and methods

An expanded “Materials and methods” section containing details regarding the isolation, culture, purification, and characterization of PBMSCs; cell treatments and groups; analysis of cell proliferation and apoptosis; enzyme-linked immunosorbent assays (ELISAs); quantitative real-time reverse transcription-PCR (qRT‒PCR); immunoblotting; immunocytofluorescence; fluorescence-activated cell sorting (FACS); animal allocation; the animal model; the study design; gene transfer; cell transplantation; echocardiography; histology; terminal deoxynucleotidyl transferase nick-end-labeling (TUNEL) staining analysis; and statistics is available in the Online Data Supplement.

### Antibodies and reagents

Supplementary Table 1 and Supplementary Table 2 list the primer sequences and the antibodies used to analyze the mRNA and protein levels, respectively. We purchased 4′,6-diamidino-2-phenylindole (DAPI, catalog 28718-90-3) from Sigma-Aldrich (St. Louis, MO, USA).

### Animals

Inbred Lewis rats were used. The Animal Care and Use Committee of GuangZhou Red Cross Hospital Medical College of Ji-Nan University approved all animal experiments (Additional file), which were carried out in compliance with the Guide for the Care and Use of Laboratory Animals published by The National Academies Press (http://www.nap.edu/).

### Isolation, expansion, and purification of PBMSCs

Rats were randomly divided into two groups (*n* = 15, each group): one group of rats underwent surgery on their backs, and the other group of rats received no surgery. PBMSCs were isolated from rat abdominal aortic blood and cultured via the adherent culture method, as described previously^12^. Fourth-generation cells were used for subsequent experiments and were assessed for purity, viability, characteristics, pluripotency, and gene transfection.

### Phenotypic characterization of PBMSCs

The characteristics of PBMSCs were determined using FACS (Becton Dickinson, Mountain View, CA, USA) to detect marker expression. The characteristics of the PBMSCs were confirmed using antibodies recognizing the MSC-associated cell surface markers CD44, SH3 [CD71], CD90, and SH2 [CD105]^13,14^; the hematopoietic stem cell (HSC) markers CD34 and CD45^14^; and the endothelial progenitor cell (EPC) molecular markers CD31 and CD133^15^. Mouse IgG1, IgG2a, and IgG2b were used as isotype controls.

### In vitro directed differentiation of PBMSCs

The PBMSCs underwent directed differentiation toward osteogenesis, chondrogenesis, adipogenesis, and angiogenesis by growth factor supplementation and growth on defined matrices. For osteogenesis, PBMSCs were induced with an osteogenic differentiation medium kit (HUXUB-90021, Cyagen, Soochow, China), and alizarin red staining was performed to evaluate osteogenic products^16^. For chondrogenesis, PBMSCs were induced using a chondrogenic differentiation medium kit (HUXUB-90041, Cyagen) and evaluated using alcian blue staining^17^. For adipogenesis, PBMSCs were cultured using an adipogenic differentiation medium kit (HUXUB-90031, Cyagen). The formation of lipid vacuoles was assessed by oil red O staining^18^. For vascular differentiation, the growth factors bFGF (5 ng/ml; Invitrogen) and vascular endothelial growth factor (VEGF) (20 ng/ml; R&D Systems; Minneapolis, MN, USA) were added. Angiogenesis was detected using immunofluorescence with double-positive staining for factor VIII and alpha smooth muscle actin (α-SMA)^11^.

### β-catenin and Oct4 transfection

Retroviral plasmid vectors, pMXs, overexpressing (*oe*) *β-catenin* (encoding β-catenin) or *Oct4* were transfected together with pReceiver-LV233 lentiviral vector (GeneCopoeia, Rockville, MD, USA) into PBMSCs using Fugene HD reagent (Fugene, Middleton, WI, USA) as directed by the manufacturer’s instructions. A pSi-LVRU6GP vector with a puromycin resistance cassette (GeneCopoeia, Rockville, MD, USA) was used to express short hairpin (*sh*) RNAs to knock down *β-catenin* and *Oct4* expression.

### Cell groups

To determine the effect of β-catenin on the proliferation and survival of PBMSCs, we randomly divided the cells into the following four groups according to different intervention methods: the no intervention group (serving as the control group), the *β-catenin* overexpression group (*oe*β-catenin), the *β-catenin* knockdown group (*sh*β-catenin), and the *β-catenin* overexpression+*β-catenin* knockdown group (*oe*β-catenin plus *sh*β-catenin). To determine the effect of Oct4 on the proliferation and survival of PBMSCs, we divided the cells into the no intervention group, the *Oct4* overexpression group (*oe*Oct4), the *Oct4* knockdown group (*sh*Oct4), and the *Oct4* overexpression+*Oct4* knockdown group (*oe*Oct4 plus *sh*Oct4). To evaluate the effects of *β-catenin* and *Oct4* on cell growth and apoptosis, we added *oe*β-cateni*n*+*sh*Oct4 and *sh*β-catenin+*oe*Oct4. After transfection, all groups of PBMSCs were cultured for 70 days for subsequent experiments to analyze the growth and apoptosis of PBMSCs.

To investigate the mechanisms of β-catenin-mediated cytoprotective effects against apoptotic cell death, we performed β-catenin activation in PBMSCs using *oe*β-catenin or a β-catenin agonist (SKL2001, MedChemexpress, Monmouth Junction, NJ, USA), and β-catenin inhibition was carried out by *sh*β-catenin or a β-catenin inhibitor (FH535, MedChemexpress).

### MTS assay

For the cell viability assay, PBMSCs were seeded at 2 × 10^3^ cells/well on a 96-well plate at 37 °C and cultured for 48 h. Cell viability was determined by the 3-(4,5-dimethylthiazol-2-yl)-5-(3-carboxymethoxyphenyl)-2-(4-sulfophenyl)-2H-tetrazolium (MTS) method in accordance with the CellTiter 96 Aqueous One Solution Viability assay manual (Promega Corporation, Madison, WI, USA).

### Growth curve of PBMSCs

PBMSCs were seeded at a density of 2 × 10^3^ cells/well onto 24-well plates in a 5% CO_2_ atmosphere at 37 °C. According to the number of cells corresponding to each time point (1, 2, 3, 4, 5, 6, and 7 d), the growth curve was drawn, and the population doubling (PD) of PBMSCs was determined.

### Colony forming assay

For each group, 500 cells were transferred to 6-well plates after transfection. A cell mass containing more than 50 cells was identified as a cell colony, and the number of cell colonies was counted under a phase contrast microscope (Olympus, Tokyo, Japan).

### Senescence-associated β-galactosidase activity

Cells on slides were sealed with parafilm and stained with X-gal staining solution (Cell Signaling Technology, Danvers, MA, USA). The stained and unstained cells were counted using a phase contrast microscope (Olympus). The percentage of senescent cells (blue) among the total cells in the field of view was calculated.

### Apoptosis detected using flow cytometry

Cells for cell cycle analysis were stained with Annexin V-APC and propidium iodide (PI) and analyzed using flow cytometry with a flow cytometer (FACSCalibur, BD Biosciences, San Jose, CA, USA). Data were analyzed using BD ModFit LT version 3.3 (BD Biosciences).

### Chromatin immunoprecipitation assays (ChIP)

Approximately 2.0 × 10^6^ cells were used in each ChIP experiment. ChIP assays were performed according to the manufacturer’s protocol from a ChIP assay kit (Merck Millipore, Billerica, MA, USA). The DNA samples were detected using real-time PCR analysis. To amplify the β-catenin binding site in the *Oct4* promoter, we used the primer sequences shown in Supplementary Table 1.

### Immunofluorescence staining

Cells or tissues were fixed with 4% paraformaldehyde (Sigma). Immunofluorescence staining was performed by incubation with antibodies recognizing β-catenin, Oct4, Factor VIII, α-SMA, Angiopoietin 1 (Ang1), or VEGF (Supplementary Table 2).

### Quantitative real-time reverse transcription PCR

Total mRNA was extracted according to standard protocols provided by Invitrogen (Waltham, MA, USA) and served as a template for reverse-transcription (RT)-PCR, following standard protocols from Promega. cDNA levels representing gene expression were then calculated using quantitative real-time PCR. The primer sequences used are shown in Supplementary Table 1. The expression of all genes was calculated using the 2^−ΔΔCt^ method using *Gapdh* (encoding glyceraldehyde-3-phosphate dehydrogenase) as the control^19^.

### Western blotting

Cells or hearts were collected and pulverized to extract protein for immunoblotting. Supplementary Table 2 lists the antibodies used to analyze the levels of the transcription factors or cytokines.

### EGFP labeling

At 24 h after transfection with the *oe*β-catenin, *sh*β-catenin, *oe*Oct4, *sh*Oct4, or control vectors, cells were cotransfected with a lentiviral vector containing enhanced green fluorescent protein (EGFP) cDNA, as described previously^20^. More than 70% of PBMSCs were GFP-positive, as determined by flow cytometry.

### Rat MI model, cell therapy, and groups

Inbred Lewis rats were used. After anesthesia with an intraperitoneal injection of 3% sodium pentobarbital, MI was induced in the rats by ligating the left anterior descending coronary artery. Animals with an ejection fraction (EF) <70% and fractional shortening (FS) <35%, as evaluated using echocardiography after induction of MI, were selected. After establishment of the MI model, the animals randomly received an injection of phosphate-buffered saline (PBS) or PBMSCs pretreated with *oe*β-catenin, *sh*β-catenin, *oe*Oct4, *sh*Oct4, or control vectors. Cell transplantation was achieved by injection into the infarct and peri-infarct regions (5 × 10^6^ cells, four sites, two sites per infarct or peri-infarct area, 20 ml per site, 1–2 cm apart). To minimize postoperative pain, we sprayed 2.5% bupivacaine at the point of incision immediately before closure, and buprenorphine hydrochloride (0.03 mg/kg) was administered intramuscularly. After the final layer of skin was closed, triple antibiotic ointment (neomycin sulfate, polymyxin B sulfate, and bacitracin zinc) was applied to the wound. Cyclosporin A (Novartis Pharma, Basel, Switzerland) was administered daily (5 mg/kg, i.h. [subcutaneous injection]) from the first day after MI until the animals were sacrificed on Day 90. Finally, twenty animals were studied in each subgroup.

### Echocardiography

Under general anesthesia, as described above, rats underwent echocardiography in a 7.5-MHz phased-array transducer (Acuson Sequoia 256, Siemens, Malvern, PA, USA) operated by an experienced technician blinded to the treatment group identity. Two-dimensional images were obtained at the midpapillary and apical levels. The left ventricular end-diastolic volume (LVEDV) and internal diameter at diastolic phase (LVEDD) were measured using the biplane area-length method. LV FS was calculated according to the modified Simpson method: FS (%) = [(LVIDd-LVIDs)/LVIDd] × 100, where LVID is the LV internal dimension, s is systole, and d is diastole. All measurements were averaged for three consecutive cardiac cycles.

### Histology

At the end of each study (90 days after cell transplantation), rats were sacrificed by cervical dislocation, and then, the hearts were perfused with 4% buffered formalin, harvested, and sectioned into 2 transverse slices parallel to the atrioventricular ring. Myocardial tissue sections were randomly chosen from five animals for triphenyltetrazolium chloride (TTC) staining. The infarct size was determined by calculating the percentage of the infarcted area against the whole LV area using Scion ImageJ (NIH, Bethesda, MD, USA). The peri-infarct regions from the MI model rats and cell therapy rats were embedded in paraffin, sectioned, and stained with Masson trichrome staining, hematoxylin and eosin (H&E), and immunofluoroscence. In each case, five independent images from each area were analyzed from each section.

### Enzyme-linked immunosorbent assays (ELISAs)

Blood samples were collected from rats by cardiac puncture under anesthesia. The levels of Ang1, bFGF, hepatocyte growth factor (HGF), and VEGF in the supernatant of heart tissues and the plasma of rats were measured using an ELISA kit according to the manufacturer’s instructions.

### Engraftment and vasculogenesis

Heart tissues were immunostained for EGFP-PBMSCs, and the cardiomyocyte marker protein troponin T was detected to accurately verify the retention of transplanted PBMSCs. Cells were collected from the left ventricles of five randomly selected hearts per experimental group, as described previously^11^. The collected cells were washed with PBS and analyzed with flow cytometry (Becton Dickinson). Engraftment was evaluated by determining the proportion of cells that expressed EGFP relative to all isolated ventricular cells. Vascular differentiation was evaluated by calculating the proportion of cells that expressed both EGFP and CD31 relative to all EGFP-positive cells. Immunofluorescence staining was performed to evaluate the expression levels of factor VIII, Ang1, and VEGF on all EGFP-positive cells using the appropriate antibodies.

### Statistical analysis

Data are presented as the mean ± standard error of the mean (SEM). Discrete variables are presented as the frequency and proportion. After performing a normality test (Shapiro–Wilk test) and homogeneity test of variance, we used the data that satisfied a normal distribution and equal variance assumptions for one-way analysis of variance (ANOVA) of these variables. When the data conformed to a normal distribution but had nonhomogeneity of variance, Welch ANOVA was performed. Comparisons were performed using the chi-squared or Fisher’s exact test for discrete variables. A 95% confidence interval (CI) (*P* < 0.05) was considered significant.

## Results

### Characterization of PBMSCs

**Fig. 1:**
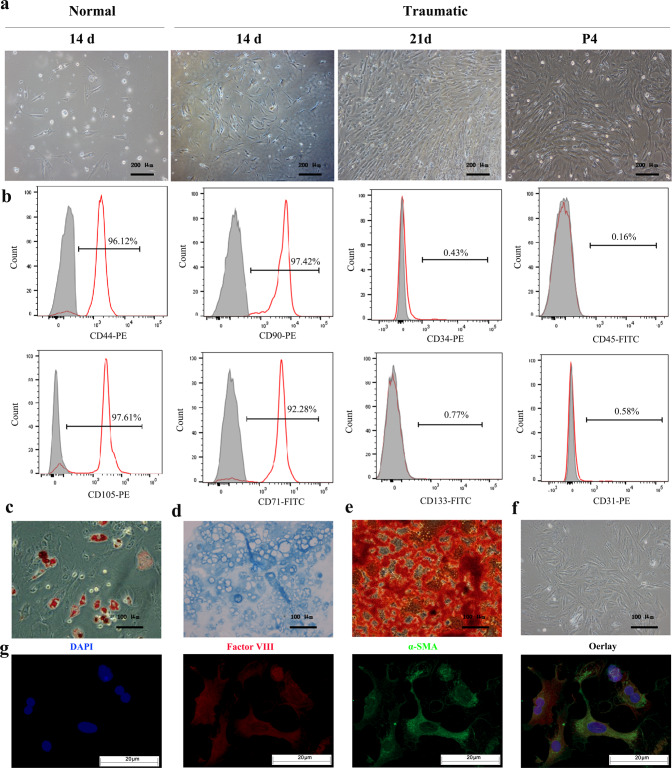
Characterization of PBMSCs. **a** Representative images of primary cultures of rat PBMSCs from normal rats at Day 14 and traumatized rats at Day 14 and Day 21 and the fourth passage of PBMSCs. Scale bars = 200 µm. **b** Flow cytometry analysis of PBMSC-positive surface markers for the MSC markers CD44, CD71, CD90, and CD105 and negative markers for the HSC markers CD34 and CD45 and EPC markers CD31 and CD133. **c**–**g** PBMSCs were induced to differentiate into adipocytes, chondrocytes, osteoblasts, and vascular cells. **c** Adipogenesis-committed differentiation of PBMSCs stained by oil red O (red). **d** Chondrogenesis-committed differentiation of PBMSCs stained with alcian blue (blue). **e** Osteogenesis-committed differentiation of PBMSCs stained by alizarin red (red). **f** Staining after normal culture with a dye-free solution. Scale bars = 100 µm. **g** Vascular endothelial cells were examined via immunofluorescence for the expression of the ectodermal cell markers Factor VIII (red) and α-SMA (green). DAPI staining (nuclei; blue) and merged images are also shown. Scale bars = 20 µm.

Adherent PBMSCs were observed after 24 h in primary cultures. The adherent PBMSCs appeared spindle-shaped in the first 7 days. Thereafter, polygonal or fibroid cells appeared. Cell colonies formed 14 days later, and the bottom of the flask was completely covered by PBMSCs at approximately 21 days. After being subcultured every 3 days, adherent cells of the fourth passage (P4) had a homogeneous and fibroblast-like morphology (Fig. 1a) and were used in subsequent experiments. Compared with those from traumatized rats, PBMSCs from the abdominal aortic blood of normal rats showed very low proliferation, suggesting a very low recovery of MSCs from normal peripheral blood (PB). Thus, we obtained PBMSCs from the peripheral blood of the traumatized rats. Immunophenotypic analyses by flow cytometry indicated that the P4 PBMSCs were strongly positive for the MSC-associated cell surface markers CD44 (96.12%), SH2 (CD105, 97.61%), CD90 (97.42%), and SH3 (CD71, 92.28%) but negative for the HSC markers CD34 (0.43%) and CD45 (0.16%) and EPC molecular markers CD133 (0.77%) and CD31 (0.58%) (Fig. 1b).

To perform a more comprehensive analysis of the multipotent differentiation of PBMSCs, we performed direct differentiation toward adipocytes, chondrocytes, osteoblasts, and vascular cells by growth factor supplementation and growth on defined matrices. PBMSCs cultured under adipogenic conditions presented cytoplasmic lipid droplets under light microscopy. The culture was stained with oil red O to confirm that the contents of the droplets were lipids (Fig. 1c). Aggrecan expressed in chondrogenesis-induced cells was demonstrated by alcian blue staining (Fig. 1d). Alizarin red-stained, nodule-like aggregations appeared in the osteogenic media on the 21st day of culture (Fig. 1e), and the negative staining results of the multiple differentiation potential of PBMSCs are shown in Fig. 1f. Immunofluorescence showed that PBMSCs positively coexpressed the blood vascular markers factor VIII and α-SMA (Fig. 1g). These results suggest that PBMSCs have the inherent characteristics of MSCs.

### β-catenin promotes PBMSC vitality and long-term survival

**Fig. 2:**
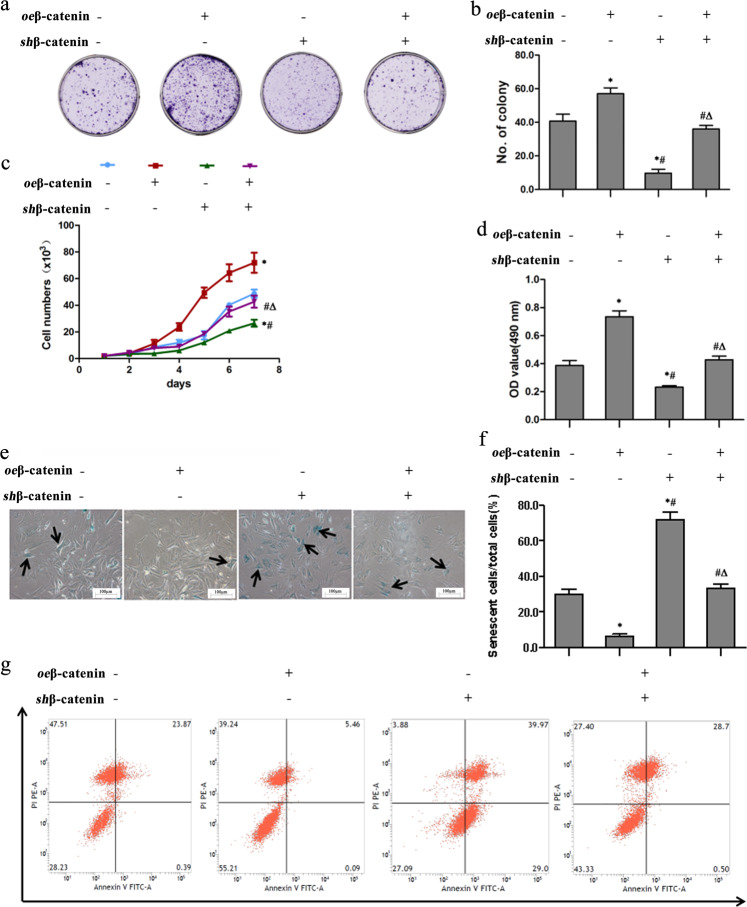
β-catenin promotes PBMSC vitality and long-term survival. The experiments were divided into the following groups: transfection of vehicle (-), lentivirus vector encoding β-catenin (*oe*β-catenin), β-catenin shRNA (*sh*β-catenin), and β-catenin or siRNA (*oe*β-catenin plus *sh*β-catenin). **a** The ability of all groups of PBMSCs to form clones was tested by cell colony formation assays and crystal violet staining. **b** Quantitative analysis of the number of cell clones in (**a**). **c** The growth curve of all groups of PBMSCs at 1, 2, 3, 4, 5, 6, and 7 d. **d** The viability of PBMSCs after 70 days of culture was tested by the MTS method. **e** β-Galactosidase staining (black arrowheads) was used to investigate cellular senescence in all groups of PBMSCs. Scale bars = 100 µm. **f** Quantitative analysis of the ratio of senescent PBMSCs to total PBMSCs in (**e**). **g** The apoptosis level of all groups of PBMSCs was assessed by flow cytometry using Annexin V/propidium iodide (PI) staining. All graphical data show the means ± SEMs. *P* < 0.05: * *vs*. the vehicle group, *# vs*. the *oe*β-catenin group, Δ *vs*. the *sh*β-catenin group, *n* = 10 in each group.

To assess the function of β-catenin, we transfected PBMSCs with a lentiviral vector encoding β-catenin (*oe*β-catenin), β-catenin shRNA (*sh*β-catenin), or *oe*β-catenin plus *sh*β-catenin or vehicle (control). After 7 days of culture, the colony number in the *oe*β-catenin-treated PBMSCs was 1.5-fold and 1.8-fold greater than that in the vehicle- and the *sh*β-catenin-treated PBMSCs, respectively (Fig. 2a, b). However, no significant difference was observed between the PBMSCs treated with *oe*β-catenin plus *sh*β-catenin or vehicle. A proliferation curve was used to assess PBMSC growth. For all groups of PBMSCs, the number of cells was ~4.0 × 10^3^ after 2 days of culture, showing no significant difference among the groups. However, after 7 days of culture, the cell number in the *oe*β-catenin-treated PBMSCs increased to 73.3 × 10^3^ and was significantly greater than that in the PBMSCs treated with *oe*β-catenin plus *sh*β-catenin or vehicle. However, the growth curves of the PBMSCs treated with *oe*β-catenin plus *sh*β-catenin and the PBMSCs treated with vehicle exhibited a similar pattern (Fig. 2c). After 70 days of culture, the viability of PBMSCs was determined by MTS experiments. The OD value was significantly higher in the *oe*β-catenin-treated PBMSCs than in the PBMSCs treated with *oe*β-catenin plus *sh*β-catenin or vehicle (Fig. 2d).

To investigate the effects of β-catenin on cell cycle arrest in PBMSCs, we performed senescence-associated (SA)-β-galactosidase (SA-β-Gal) staining. The number of SA-β-Gal-stained cells was markedly smaller in the *oe*β-catenin-treated group than in the *oe*β-catenin plus *sh*β-catenin or vehicle groups (Fig. 2e). The ratio of senescent/total cells showed a significant decrease in the *oe*β-catenin-treated group compared with the *oe*β-catenin plus *sh*β-catenin or vehicle groups. In contrast, compared with that in the vehicle group, *β-catenin* knockdown resulted in a more pronounced increase in the ratio of senescent/total cells in the *sh*β-catenin group, and no significant change was observed in the *oe*β-catenin plus *sh*β-catenin group (Fig. 2f). The antiapoptotic effect of β-catenin on PBMSCs was evaluated using Annexin V-APC and PI staining, followed by flow cytometry. The proportion of Annexin V-positive cells was lower in the *oe*β-catenin group than in the vehicle group but dramatically higher in the *sh*β-catenin group than in the vehicle group (Fig. 2g). However, there was no significant difference in apoptotic rates between the PBMSCs treated with *oe*β-catenin plus *sh*β-catenin and the vehicle-treated PBMSCs. These results revealed that β-catenin promotes PBMSC vitality and long-term survival.

### Oct4 promotes PBMSC vitality and long-term survival

We next investigated the mechanisms by which β-catenin regulates PBMSC survival under hypoxia. Oct4, a homeobox transcription factor (TF), is essential for bone marrow MSC self-renewal^21^. To determine the underlying mechanism of Oct4 in β-catenin-mediated survival of PBMSCs, we analyzed key TFs related to stem cell stemness. We found that β-catenin overexpression markedly increased *Oct4* mRNA expression in the *oe*β-catenin-treated PBMSCs, whereas *Oct4* mRNA was almost undetectable in the β-catenin-deficient PBMSCs (Supplementary Fig. 1a). Moreover, Oct4 protein levels were markedly reduced in the β-catenin-deficient PBMSCs (Supplementary Fig. 1b). Immunofluorescence revealed that Oct4 expression was consistent with the expression of β-catenin (Supplementary Fig. 1c). However, alteration of β-catenin expression did not cause a significant change in the expression of c-Myc, Nanog, and Kruppel-like factor 4 (KLF4), suggesting that Oct4 is the primary regulator of these stemness factors, mediated by β-catenin in PBMSCs.

**Fig. 3:**
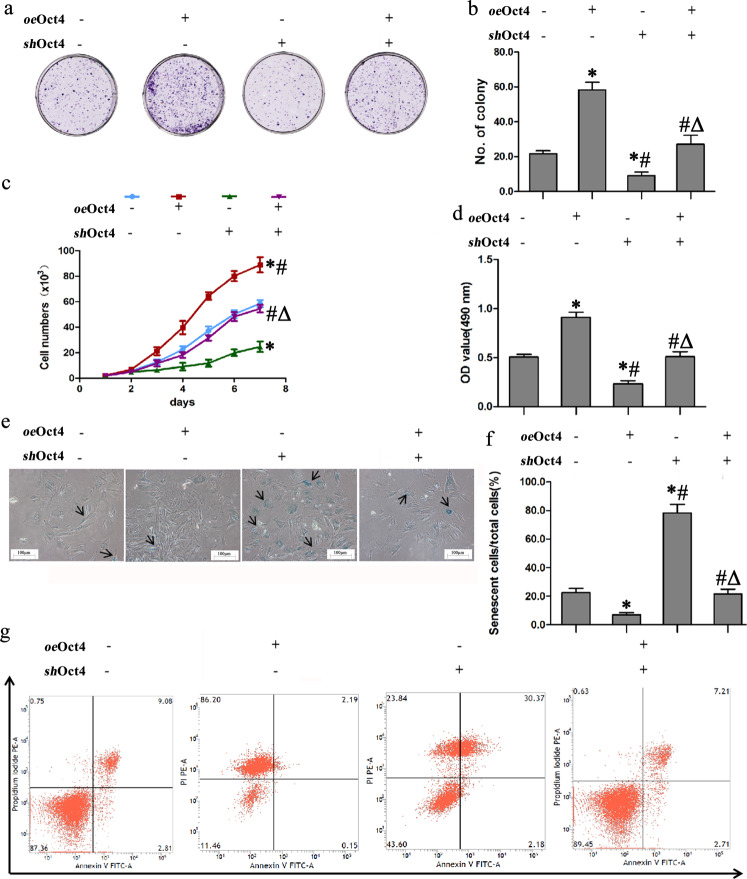
Oct4 promotes PBMSC vitality and long-term survival. **a** The ability of all groups of PBMSCs to form clones was tested by cell colony formation assays and crystal violet staining. **b** Quantitative analysis of the number of cell clones in (**a**). **c** The growth curve of all groups of PBMSCs at 1, 2, 3, 4, 5, 6, and 7 d. **d** The viability of PBMSCs after 70 days of culture was tested by the MTS method. **e** Cell morphology senescence-associated β-galactosidase staining (black arrowheads) in PBMSCs from all groups. Scale bars = 100 µm. **f** Quantitative analysis of the ratio of senescent PBMSCs to total PBMSCs in (**e**). **g** The apoptosis level of all groups of PBMSCs was detected by flow cytometry using Annexin V/PI staining. All graphical data are shown as the means ± SEMs. *P* < 0.05: * *vs*. the vehicle group, *# vs*. the *oe*β-catenin group, *Δ vs*. the *sh*β-catenin group, *n* = 10 in each group.

We next determined whether Oct4 regulates the vitality and survival of long-term cultured PBMSCs. PBMSCs were transfected with a lentivirus overexpressing Oct4 (*oe*Oct4) or Oct4 shRNA (*sh*Oct4) or vehicle or cotransfected with *oe*Oct4 plus *sh*Oct4. After 7 days of culture, we observed that Oct4 overexpression markedly increased colony formation in the *oe*Oct4 group and *Oct4* knockdown significantly decreased colony formation in the *sh*Oct4 group compared with the vehicle group (Fig. 3a). Statistical analysis in comparison with the vehicle group showed that the colony number increased by 2.1-fold in the *oe*Oct4 groups and decreased by 1.3-fold in the *sh*Oct4 group (Fig. 3b). The proliferation curve was drawn to determine PBMSC growth. All cells grew and reached ~4.0 × 10^3^ at 2 days of culture, and there were no significant differences in the number of cells among the groups. After 7 days of culture, the number of cells increased to 9.0 × 10^4^ in the *oe*Oct4 group but decreased to 2.6 × 10^4^ in the *sh*Oct4 group. Moreover, the growth curves of the PBMSCs treated with *oe*Oct4 plus *sh*Oct4 or vehicle exhibited a similar pattern (Fig. 3c). When PBMSCs were cultured for 70 days, the OD value was significantly higher in the *oe*Oct4 group than in the vehicle group, while the OD value was significantly lower in the *sh*Oct4 group than in the vehicle group. However, there was no significant difference in the OD value between the *oe*Oct4 plus *sh*Oct4 and vehicle groups (Fig. 3d).

The effects of Oct4 on the senescence activity in PBMSCs were also measured using the SA-β-Gal staining kit. As expected, the *oe*Oct4 group presented lower SA/β-Gal activity than the *sh*Oct4 and vehicle groups (Fig. 3e). Quantitative analysis showed that compared with vehicle treatment, Oct4 overexpression significantly decreased SA-β-Gal staining, and Oct4 deficiency increased it (Fig. 3f). The number of senescent cells was not significantly different between the vehicle and *oe*Oct4 plus *sh*Oct4 groups. After 70 days of culture, ~9% of the vehicle-treated PBMSCs underwent apoptosis (Fig. 3g). Oct4 overexpression caused a significant decrease in the apoptosis rate, while *Oct4* knockdown significantly increased the rate. Moreover, there was no significant difference in the apoptotic ratio between the vehicle and *oe*Oct4 plus *sh*Oct4 groups. These results revealed that Oct4 promotes PBMSC vitality and long-term survival.

### β-catenin regulates *Oct4* transcription in PBMSCs

**Fig. 4:**
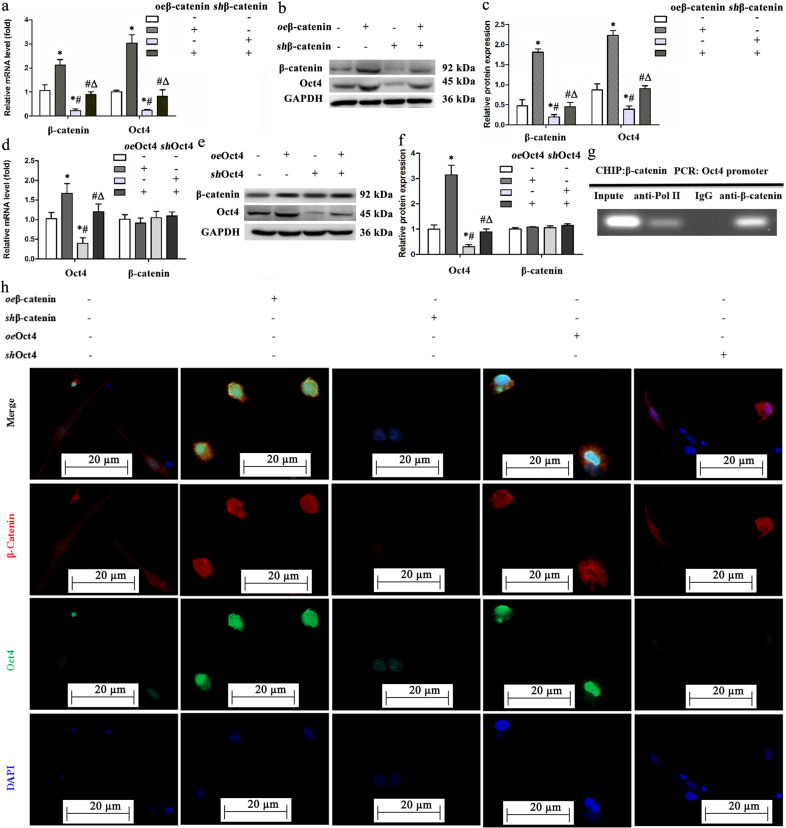
β-catenin initiates Oct4 activation. **a**–**c** β-catenin overexpression increases the mRNA and protein levels of β-catenin and Oct4. Their mRNA expression levels were measured by qRT‒PCR (**a**). The protein levels of β-catenin and Oct4 were then detected using western blotting (**b**, **c**). **d**–**f** The mRNA and protein levels of β-catenin and Oct4 were measured by qRT‒PCR (**d**) and immunoblotting (**e** and **f**) in PBMSCs treated with vehicle, *oe*Oct4, *sh*Oct4, or *oe*Oct4 plus *sh*Oct4. **g** Chromatin immunoprecipitation (ChIP) assay for the binding of β-catenin to the *Oct4* promoter. Anti-IgG was used as a negative control, and anti-RNA-polymerase II was used as a positive control. **h** Representative immunostaining in the PBMSCs treated with vehicle, *oe*Oct4, *sh*Oct4, *oe*Oct4, or *sh*Oct4 showing that β-catenin overexpression increases the expression of both β-catenin and Oct4, but alteration of *Oct4* expression did not significantly change β-catenin expression. β-Catenin or Oct4 silencing markedly suppressed the expression of Oct4. β-Catenin and Oct4 were stained red and green, respectively. The nuclei of PBMSCs were counterstained blue using 4,6-diamino-2-phenyl indole (DAPI). Scale bars = 20 µm. β-Catenin was mainly expressed in the cytoplasm and partially in the cell nucleus. Oct4 was mainly localized in the nucleus. All graphical data are shown as the means ± SEMs. *P* < 0.05: * *vs*. the vehicle group, *# vs*. the *oe*β-catenin group, *Δ vs*. the *sh*β-catenin group, *n* = 10 for each group.

Next, we determined the relationship between β-catenin and Oct4. Figure 4a–c shows that compared with those in the vehicle group, the mRNA and protein levels of β-catenin and Oct4 were significantly increased by β-catenin overexpression, decreased by β-catenin inhibition, and showed no significant change in the PBMSCs treated with *oe*β-catenin plus *sh*β-catenin. Although Oct4 overexpression or deficiency led to increased or decreased mRNA and protein levels of Oct4, the mRNA and protein expression levels of β-catenin showed no significant difference among the vehicle, *oe*Oct4, *sh*Oct4, and *oe*Oct4 plus *sh*Oct4 groups (Fig. 4d–f). Immunofluorescently stained PBMSCs showed similar change patterns: (1) The fluorescence intensity of β-catenin and Oct4 was stronger in the *oe*β-catenin group than in the vehicle group but was significantly decreased in the *sh*β-catenin group; (2) overexpression of Oct4 led to a substantial increase in Oct4 expression, while β-catenin expression was similar in the groups receiving *oe*Oct4 or *sh*Oct4 (Fig. 4h). To confirm the binding of β-catenin to the *Oct4* promoter, we performed a ChIP assay of PBMSCs. The results suggested that *Oct4* promoter fragments could only be amplified from chromatin precipitated using anti-β-catenin antibodies (Fig. 4g). All these results suggested that β-catenin regulates the expression of Oct4 by directly binding an Oct4 regulatory region, while Oct4 has no regulatory effect on β-catenin in PBMSCs.

### β-catenin regulates Oct4-related antiapoptotic and proangiogenic signaling pathways

To provide further mechanistic insights into β-catenin/Oct4-mediated cytoprotective effects against apoptotic cell death, we assessed antiapoptotic and proapoptotic molecules. First, PBMSCs were treated with a β-catenin agonist or inhibitor. The addition of the β-catenin agonist to the culture media strongly enhanced the expression of β-catenin, Oct4, and the antiapoptotic molecules bFGF, survivin, and Bcl2 and decreased the expression of the proapoptotic molecules Bax and caspase-3. Suppression of β-catenin by its inhibitor had the opposite effects in PBMSCs. However, the β-catenin agonist or inhibitor did not significantly change glycogen synthase kinase 3 beta (GSK3β) expression (Supplementary Fig. 2a, b). These data strongly suggested that activation of Wnt/β-catenin signaling mainly upregulated β-catenin/Oct4-specific antiapoptotic protein expression and downregulated the expression of proapoptotic molecules but only slightly affected GSK-3β signaling. Thus, we next performed direct gain- or loss-of-function experiments to specifically evaluate the effects of β-catenin and Oct4 on the expression of these antiapoptotic or proapoptotic molecules by transfecting the cells with vectors encoding β-catenin/Oct4 (*oe*β-catenin/*oe*Oct4) or lentiviruses carrying targeted β-catenin/Oct4 shRNA (*sh*β-catenin/*sh*Oct4).

**Fig. 5:**
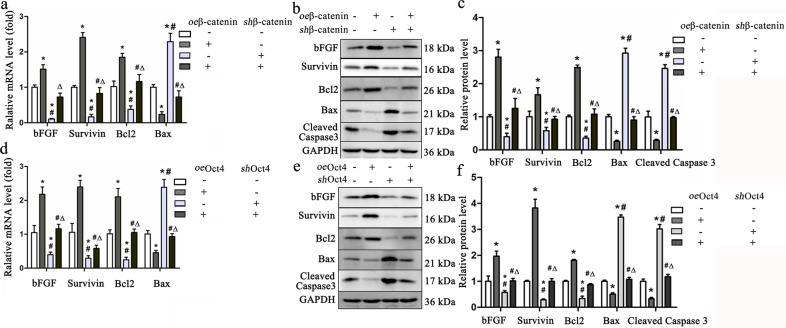
β-catenin/Oct4 regulates the expression of antiapoptotic molecules. **a** The mRNA and protein levels of bFGF, survivin, Bcl2, Bax (**a**–**c**), and cleaved caspase-3 (**b**, **c**) in the PBMSCs treated with vehicle, *oe*β-catenin, *sh*β-catenin, or *oe*β-catenin plus *sh*β-catenin were evaluated using qRT‒PCR (**a**) and western blotting (**b**, **c**). **d**–**f** The mRNA and protein levels of these molecules from PBMSCs treated with vehicle, *oe*Oct4, *sh*Oct4, or *oe*Oct4 plus *sh*Oct4 were also detected using qRT‒PCR (**d**) and western blotting (**e**, **f**). All graphical data are shown as the means ± SEMs. *P* < 0.05: * *vs*. the vehicle group, # *vs*. the *oe*β-catenin group, *Δ vs*. the *sh*β-catenin group, *n* = 10 for each group.

Gain- and loss-of-function experiments demonstrated that the mRNA and protein levels of the proapoptotic molecules Bax and cleaved caspase-3 were lower in the *oe*β-catenin-treated PBMSCs than in the vehicle-treated PBMSCs and higher in the *sh*β-catenin-treated PBMSCs than in the vehicle-treated PBMSCs (Fig. 5a–c). In contrast, β-catenin overexpression increased the mRNA and protein levels of the antiapoptotic molecules Bcl2 and survivin and the proangiogenic cytokine bFGF in the *oe*β-catenin-treated PBMSCs, while β-catenin deletion inhibited their expression. This cytoprotection was further confirmed using Oct4 overexpression, which promoted the mRNA and protein levels of bFGF, Bcl2, and survivin and inhibited Bax and cleaved caspase-3 levels in the *oe*β-catenin-treated PBMSCs, all of which were markedly abolished in the *sh*β-catenin-treated PBMSCs (Fig. 5d–f). However, the levels of all these molecules in the PBMSCs treated with *oe*β-catenin plus *sh*β-catenin were comparable to those in the vehicle-treated PBMSCs. Moreover, gain and loss of function of β-catenin and Oct4 were involved in the growth and apoptosis of PBMSCs. PBMSCs cultured with vehicle gradually grew slowly, showing a 2-fold increase in PD on the 2nd-day post-culture, and cell doubling increased slowly, being only maintained for 4 days, and then gradually decreased on the 5th day. When transfected with β-catenin or Oct4 expression vectors, PBMSCs showed an over 2-fold increase in PD in less than 2 days, and cell doubling increased up to 7 d. Silencing of *β-catenin* or *Oct4* markedly reduced cell growth. This reduction was rescued by Oct4 overexpression (*sh*β-catenin+*oe*Oct4, Supplementary Fig. 3a). This phenomenon was consistent with antiapoptotic effects. Overexpression of β-catenin or Oct4 significantly attenuated cell apoptosis; however, silencing of *β-catenin* or *Oct4* abolished this benefit. There was no significant difference in the apoptotic rates between the PBMSCs treated with *sh*β-catenin, *oe*β-catenin+*sh*Oct4, and *sh*Oct4. Conversely, the proportion of Annexin V-positive cells was lower in the *oe*β-catenin, *sh*β-catenin+*oe*Oct4, and *oe*Oct4 groups than in the vehicle group but was markedly higher in the *sh*β-catenin and *sh*Oct4 groups than in the vehicle group (Supplementary Fig. 3b, c). However, there was no significant difference in apoptotic rates between the *oe*β-catenin+*sh*Oct4- and vehicle-treated PBMSCs. Taken together, these findings demonstrated that β-catenin/Oct4 signaling regulates the expression of apoptosis-related genes to maintain long-term cell survival.

### β-catenin/Oct4 augments myocardial repair induced by PBMSC therapy

**Fig. 6:**
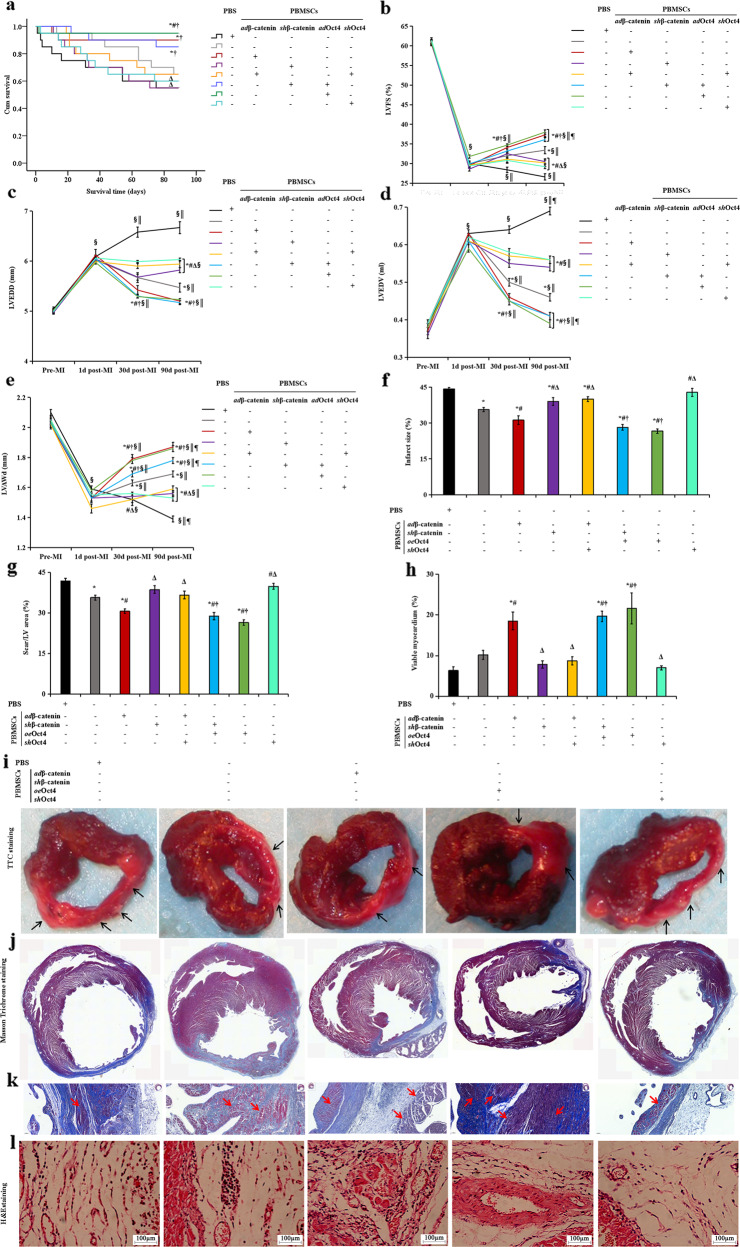
β-catenin functionally regulates myocardial repair of PBMSCs in infarcted hearts through direct targeting of Oct4. **a** Kaplan–Meier survival rates. **b**–**e** Echocardiography of LVFS (**b**), LVEDD (**c**), LVEDV (**d**), and LVAWd (**e**) before MI (pre-MI, baseline), 1 d after MI (1 d post-MI), 30 d after MI (30 d post-MI) and 90 d after MI (90 d post-MI). Transplantation of the PBMSCs pretreated with β-catenin or Oct4 transfection had higher survival rates and resulted in significant improvement in cardiac function and structural remodeling. However, this effect was abolished in the *sh*β-catenin or *sh*Oct4 groups (*oe*β-catenin+*sh*Oct4) and was rescued by combination with Oct4 transfection (*sh*β-catenin+*oe*Oct4). For determination of the effects of the *oe*β-catenin- or *oe*Oct4-transfected MSCs on left ventricle structure at 90 days after MI, hearts were stained with TTC, Masson’s trichrome, or hematoxylin and eosin. **f** Quantitation of infarct size as determined by TTC staining at 90 d post-MI. **g** Morphometric measurements of the scar/LV area revealed a smaller scar in the *oe*β-catenin- or *oe*Oct4-transfected PBMSC-treated groups and a larger scar in the *sh*β-catenin- or *sh*Oct4-transfected PBMSC-treated groups than in the PBMSC- and PBS-treated groups. **h** Morphometric measurements of the viable cardiomyocytes in rat hearts from the different groups showed significantly larger areas of cardiomyocytes in the hearts treated with *oe*β-catenin or *oe*Oct4-transfected PBMSCs than with PBMSCs alone, *sh*β-catenin or *sh*Oct4-transfected PBMSCs and PBS. **i** Representative histological images of left ventricular basal, middle, and apex cross-sections after TTC staining in the PBS, PBMSC, and *oe*β-catenin-, *oe*Oct4- or *sh*Oct4-transfected PBMC groups. TTC-stained images were cut into transverse sections to assess the infarct size (percentage of the area of the entire LV, **f**). The surviving myocardium was stained brick red by TTC staining, and the infarcted myocardium was pale white (black arrows). In the *oe*β-catenin- or *oe*Oct4-transfected PBMC groups, the ischemic area was significantly smaller than that in the PBS and *sh*Oct4-transfected PBMC groups, suggesting a positive effect of β-catenin/Oct4-overexpressing PBMSCs on the infarcted hearts. **j**, **k** Collagen density measured by Masson’s trichrome staining was lower in the *oe*β-catenin- or *oe*Oct4-transfected PBMC-treated animals after 90 days. The infarct zone of the *oe*β-catenin- or *oe*Oct4-transfected PBMC-treated groups contained islands of viable cardiomyocytes (red, **k**, red arrows, Label, 50 μm). **l** H&E staining showing different levels of fibrocyte infiltration and inflammation in the ischemic area. Label, 100 μm. All graphical data are shown as the means ± SEMs. *P* < 0.05: *vs. PBS injection, ^#^*vs*. PBMSC therapy alone, ^Δ^*vs*. transplantation of *oe*β-catenin, *oe*Oct4-transfected PBMSCs, or *sh*β-catenin plus *oe*Oct4, ^†^*vs*. transplantation of PBMSCs transfected with *oe*β-catenin plus *sh*Oct4 or *sh*β-catenin alone, ^§^*vs*. pre-MI, ^║^*vs*. 1d post-MI, ^¶^*vs*. 30 d post-MI. **b**–**e** Pre-MI and 1 d post-MI, *n* = 20 per group. At 30 d post-MI, PBS injection, *n* = 15; cell therapy: PBMSCs alone, *n* = 18; *oe*β-catenin-treated PBMSCs, *n* = 18; *sh*β-catenin-treated PBMSCs, *n* = 16; PBMSCs treated with *oe*β-catenin plus *sh*Oct4, *n* = 16; PBMSCs treated with *sh*β-catenin plus *oe*Oct4, *n* = 19; PBMSCs treated with *oe*Oct4, *n* = 19; and PBMSCs treated with *sh*Oct4, *n* = 16. At 90 d post-MI, PBS injection, *n* = 11; cell therapy: PBMSCs alone, *n* = 12; *oe*β-catenin-treated PBMSCs, *n* = 18; *sh*β-catenin-treated PBMSCs, *n* = 11; PBMSCs treated with *oe*β-catenin plus *sh*Oct4, *n* = 13; PBMSCs treated with *sh*β-catenin plus *oe*Oct4, *n* = 17; PBMSCs treated with *oe*Oct4, *n* = 19; and PBMSCs treated with *sh*Oct4, *n* = 12. (**f**–**h**, *n* = 5, each group).

Next, we investigated whether the β-catenin/Oct4 signaling pathway augmented PBMSC engraftment, survival, and functional recovery after MI induction. We subjected rats to MI by ligation of the left anterior descending coronary artery. After pretreatment of PBMSCs transfected with *oe*β-catenin, *sh*β-catenin, or vehicle (–) or in combination with *sh*Oct4 or *oe*Oct4 lentiviral vectors, only *sh*Oct4 or only *oe*Oct4 was randomly transplanted into the infarcted hearts. The rats with MI were also randomly assigned to receive PBS injection as a control group. The 160 animals were randomly divided into eight groups and underwent serial echocardiography studies. Thereafter, all animals were followed-up for 90 days, during which 46 rats died. The surviving 114 rats were then sacrificed and subjected to pathology and molecular biology tests. After 90 days, Kaplan–Meier survival analysis showed a higher survival rate in the rats receiving *oe*β-catenin- or *oe*Oct4-treated PBMSC transplantation than in the rats receiving PBS injection and PBMSC therapy alone (90%/95% in the rats receiving *oe*β-catenin/*oe*Oct4-treated PBMSCs *versus* 55% in the rats receiving PBS, *P* = 0.017/0.005; *versus* 65% in the rats receiving PBMSCs alone, *P* = 0.091/0.026), and this effect was eliminated in the rats receiving *sh*β-catenin or *sh*Oct4. Moreover, the inhibition resulting from *β-catenin* knockdown was rescued by Oct4 overexpression (*sh*β-catenin+*oe*Oct4). However, no significant differences were found between the animals receiving *oe*β-catenin- or *oe*Oct4-treated PBMSCs and between those receiving PBMSCs pretreated with *sh*β-catenin or *sh*Oct4 (Fig. 6a).

Echocardiographic studies showed that on Day 1 post-infarct, the animals in all eight groups developed typical changes of acute heart failure and LV early remodeling compared with baseline levels. These changes included decreases in the cardiac function indices left ventricular fractional shortening (LVFS), dilated LVEDD and LVEDV and thinning of the left ventricular end-diastolic anterior wall thickness (LVAWd) (Fig. 6b–e). Although transplantation of PBMSCs alone transiently improved LVFS, LVEDD, LVEDV, and LVAWd at 30 d post-MI, these benefits were not sustained at 90 d post-MI. At 90 days, the rats receiving *oe*β-catenin- or *oe*Oct4-treated PBMSCs exhibited sustained improvement in LVFS, LVEDD, LVEDV, and LVAWd. These beneficial effects were eliminated in the rats receiving *sh*β-catenin or *sh*Oct4 but could be recovered by cotreatment with *oe*Oct4 (*sh*β-catenin+*oe*Oct4). The indices in the rats receiving PBS indicated sustained exacerbation, with decreased LVFS, dilation of LVEDD and LVEDV, and thinning of the LVAWd. There was no significant difference between the animals receiving *oe*β-catenin- or *oe*Oct4-treated PBMSCs and those receiving PBMSCs pretreated with *sh*β-catenin or *sh*Oct4. Thus, overexpression of β-catenin/Oct4 in PBMSCs enhanced their ability to improve cardiac remodeling after MI.

Postmortem morphometry revealed that TTC staining at Day 90 following MI showed transmural infarct scars, mostly in the apex, anteroseptal, and anterior wall in the PBS-treated rats (Fig. 6i). The infarct area was significantly smaller in the *oe*β-catenin or *oe*Oct4 groups than in the PBS group and the PBMSC alone group (Fig. 6f). Multiple sections of heart tissue were also subjected to Masson’s trichrome staining (Fig. 6j, k). Transplantation of the *oe*β-catenin- or *oe*Oct4-treated PBMSCs significantly reduced extracellular matrix deposition in the total myocardial area. The scar/LV area decreased by 30–40% in the *oe*β-catenin or *oe*Oct4 groups compared with that in the PBS group (Fig. 6g), accompanied by the greatest increase in viable cardiomyocytes in the rats receiving *oe*Oct4-treated PBMSCs (Fig. 6h). Microscopic examination of H&E staining of sections from the infarcted hearts treated with PBS injection clearly showed inflammation and fibrosis at the infarct site; however, transplantation of PBMSCs transfected with *oe*β-catenin or *oe*Oct4 caused the greatest reduction in myocardial inflammation (Fig. 6l). However, compared with PBS injection, PBMSC therapy alone did not significantly reduce the infarct size, MI-induced fibrosis, myocyte loss, or inflammation. Moreover, these beneficial effects of β-catenin were abolished by *sh*Oct4 (*oe*β-catenin+*sh*Oct4), and adding *oe*Oct4 rescued the beneficial effects (*sh*β-catenin+*oe*Oct4). Taken together, these data suggest that the β-catenin/Oct4 axis in ischemic hearts improves PBMSC-mediated restoration of cardiac function and structure.

### β-Catenin enhances the long-term survival of engrafted PBMSCs in ischemic hearts by activating Oct4-related antiapoptotic activity

**Fig. 7:**
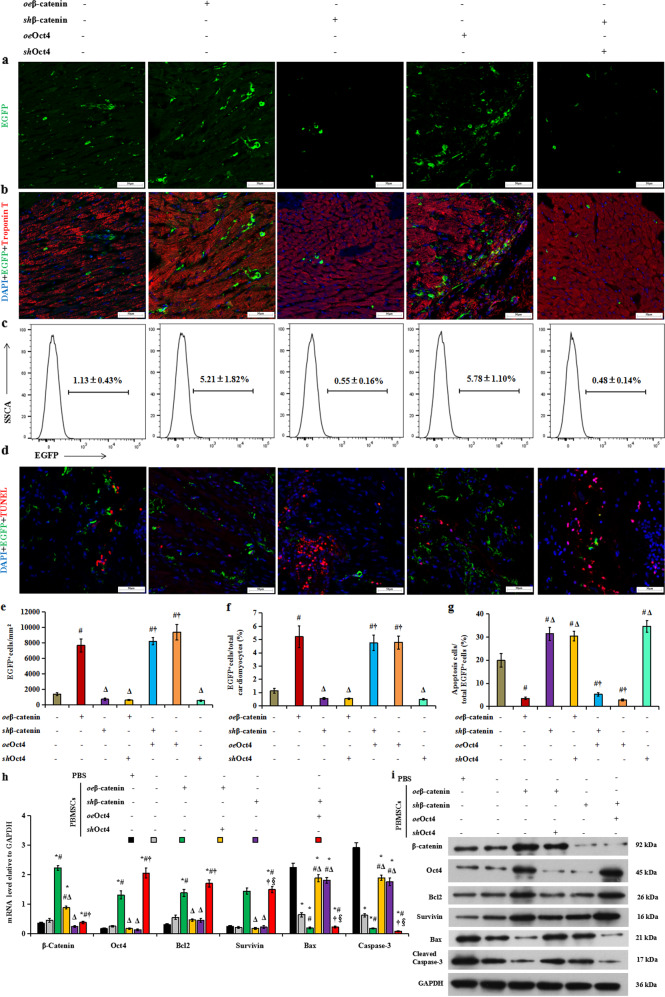
β-Catenin promotes the long-term survival of engrafted PBMSCs in ischemic hearts via the Oct4-related antiapoptotic pathway. PBMSCs were infected with a reporter retrovirus expressing EGFP before transplantation and were recognized as EGFP-positive cells. **a** Representative fluorescence microscopy images of tissue sections showing the retention of EGFP (green)-labeled PBMSCs at the injection site at 90 days after transplantation. Scale bar: 50 μm. **b** Heart tissues were immunostained for EGFP-PBMSCs (green) and the cardiomyocyte marker troponin T (red) to verify the retention of transplanted PBMSCs and were counterstained with DAPI to detect the nuclei. Overexpression of β-catenin/Oct4 markedly improved the initial retention of donor PBMSCs compared with that in vector control cells, whereas *sh*β-catenin or *sh*Oct4 decreased the retention of transplanted PBMSCs. Scale bar, 50 μm. **c** Representative phenotype of gated EGFP^+^ cells evaluated by FACS. **d** Representative double TUNEL (terminal deoxynucleotidyl transferase UTP nick-end labeling) staining for apoptotic PBMSCs in the infarcted tissues. EGFP^+^ cells are shown as green. The red fluorescence represents apoptotic cells. Merged images indicate the apoptosis of transplanted PBMSCs. Note that more EGFP-labeled PBMSCs were apoptotic in the infarcted hearts receiving *sh*β-catenin or *sh*Oct4, as revealed by the presence of cells costained with TUNEL staining (red) and EGFP (green); 4',6-diamidino-2-phenylindole (DAPI) was used as a nuclear marker. Scale bar, 50 μm. **e** Quantitative data showing the retention of EGFP^+^ PBMSCs in the ischemic hearts. **f** Quantitative analysis of the percentages of EGFP-positive cells (EGFP^+^) relative to the whole ventricular cell population in the ischemic hearts after 90 days of transplantation using FACS. **g** Quantification of the apoptosis of transplanted PBMSCs in the infarcted tissues using the TUNEL assay. All graphs show the means ± SEMs. *p* < 0.05: *vs. PBS injection, ^#^*vs*. PBMSC therapy alone, ^Δ^*vs*. transplantation of PBMSCs transfected with *oe*β-catenin, *oe*Oct4-, or *sh*β-catenin plus *oe*Oct4, ^†^*vs*. transplantation of PBMSCs transfected with *oe*β-catenin plus *sh*Oct4 or *sh*β-catenin alone (*n* = 5 per group). **h** Quantitative analysis of the mRNA expression of *β-catenin*, *Oct4*, *Bcl2*, *Birc5* (encoding survivin), *Bax*, and *Casp3* (caspase-3). **i** Representative immunoblots of these molecules. *P* < 0.05: *vs. PBS injection, ^#^*vs*. PBMSC therapy alone, ^Δ^*vs*. transplantation of *oe*β-catenin-treated MSCs, ^†^*vs*. transplantation of PBMSCs transfected with *oe*β-catenin plus *sh*Oct4, ^§^*vs*. transplantation of PBMSCs transfected with *sh*β-catenin alone (*n* = 5 per group).

We next sought to investigate whether β-catenin/Oct4 could promote the long-term survival of engrafted PBMSCs in ischemic hearts. Cell retention at 90 days after PBMSC transplantation into the infarcted hearts is shown in Fig. 7a, b. EGFP^+^ cells were highly enriched in the ischemic hearts receiving PBMSCs transfected with *oe*β-catenin or *oe*Oct4 but were sparse in the control vector-treated (−) ischemic hearts. However, β-catenin or Oct4 deficiency significantly reduced PBMSC engraftment. EGFP^+^ PBMSCs with *oe*β-catenin or *oe*Oct4 exhibited much higher engraftment in the whole infarcted heart than PBMSCs with control vectors or *sh*β-catenin (Fig. 7c). Statistical analysis showed that Oct4 deficiency abolished this benefit, and Oct4 overexpression rescued the poor engraftment of *β-catenin* knockdown PBMSCs (*sh*β-catenin+*oe*Oct4). Consistently, β-catenin transfection significantly increased the retention of EGFP^+^ PBMSCs compared with transfection with control vectors, but this effect was abolished by *Oct4* knockdown (*oe*β-catenin+*sh*Oct4, Fig. 7e, f). To assess the antiapoptotic effect of β-catenin/Oct4 on PBMSCs after MI, we performed terminal deoxynucleotidyl transferase nick-end-labeling (TUNEL) staining analysis. The results showed that compared with the control, β-catenin/Oct4 overexpression substantially decreased the percentage of apoptotic PBMSCs, whereas *sh*Oct4 significantly inhibited the antiapoptotic effect on *oe*β-catenin-induced PBMSCs (*oe*β-catenin+*sh*Oct4, Fig. 7d, g). Taken together, these data suggested that β-catenin/Oct4 could promote the engraftment of PBMSCs to prevent their loss during myocardial ischemia.

Then, we tested whether activated β-catenin/Oct4 signaling was associated with increased expression of its related apoptotic signaling molecules. Under conditions of β-catenin overexpression, the mRNA and protein levels of β-catenin, Oct4, and antiapoptotic molecules (Bcl2 and survivin) increased in cardiac tissue from the rats receiving *oe*β-catenin-treated PBMSCs. However, Oct4 deficiency inhibited this increase (*oe*β-catenin+*sh*Oct4). The levels of the proapoptotic molecules Bax and caspase-3 decreased markedly in the rats with MI that received PBMSCs pretreated with β-catenin/Oct4 overexpression (Fig. 7h, i), suggesting reduced PBMSC apoptosis induced by β-catenin/Oct4 overexpression after MI. β-Catenin inhibition completely eliminated these antiapoptotic effects, and Oct4 overexpression reversed this increase (*sh*β-catenin+*oe*Oct4). In addition, the overexpression or knockdown of β-catenin or Oct4 caused corresponding changes in the expression of Oct4, but alteration of Oct4 resulted in no significant change in β-catenin expression. All these findings suggest that β-catenin overexpression promotes the long-term engraftment of PBMSCs in ischemic hearts by activating Oct4-related antiapoptotic signaling.

### β-Catenin/Oct4 signaling enhances angiogenesis of PBMSCs in ischemic hearts

**Fig. 8:**
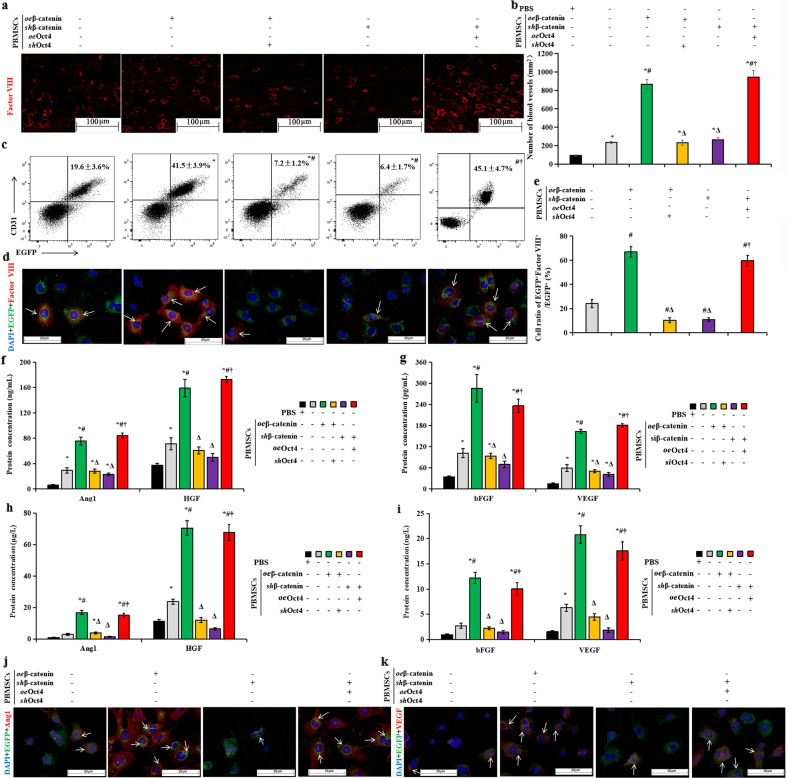
β-Catenin/Oct4 signaling promotes angiogenesis of engrafted PBMSCs in ischemic hearts. **a** Myocardial vessels were detected by immunofluorescence staining with an anti-factor VIII antibody at 90 d after transplantation of PBMSCs alone or pretreatment with *oe*β-catenin, *oe*β-catenin plus *sh*Oct4, *sh*β-catenin, or *sh*β-catenin plus *oe*Oct4. Scale bar = 100 μm. **b** Quantitative analyses of blood vessel intensity in the rats receiving cell therapy compared with that in the animals receiving PBS injection. **c** Statistical analysis of the mean percentage of CD31 and EGFP double-positive cells (CD31^+^EGFP^+^) relative to the whole EGFP^+^ population as assessed by FACS. **d** Immunofluorescence staining showing that the transplanted cells expressed factor VIII. The transplanted cells were prelabeled with EGFP (green); the nuclei were stained with DAPI (blue), and the cytoplasm of the blood endothelial cells was stained red with anti-factor VIII, scale bar = 20 μm. **e** Statistical analysis showing that engrafted EGFP-prelabeled cells expressing factor VIII were the most numerous among the PBMSCs pretreated with *oe*β-catenin, followed by cells pretreated with *sh*β-catenin plus *oe*Oct4, and *β-catenin*- or *Oct4*-silenced PBMSCs had the lowest levels (**d**, arrows). Protein concentrations of Ang1 and HGF (**f**), bFGF and VEGF (**g**) analyzed by an enzyme-linked immunosorbent assay (ELISA) in the supernatant of infarcted hearts at 90 d after treatment of PBS injection, transplantation of PBMSCs alone, or pretreatment with *oe*β-catenin, *oe*β-catenin plus *sh*Oct4, *sh*β-catenin, or *sh*β-catenin plus *oe*Oct4. The levels of these proangiogenic cytokines in the supernatant were significantly higher from the infarcted hearts receiving PBMSCs pretreated with *oe*β-catenin or *sh*β-catenin plus *oe*Oct4 than those receiving PBMSCs alone or PBMSCs pretreated with *β-catenin*- or *Oct4*-silenced cells. The changes in Ang1 and HGF (**h**) and bFGF and VEGF (**i**) were also detected by ELISAs in the plasma of these rats. Representative images of Ang1 (**j**) and VEGF (**k**) immunofluorescence staining; scale bar = 20 μm. The numbers of engrafted EGFP-prelabeled cells expressing Ang1 or VEGF were higher among the cells overexpressing β-catenin or Oct4 than in the *β-catenin*- or *Oct4*-silenced cells (white arrowheads). All graphs show the means ± SEMs. *P* < 0.05: *vs. PBS injection, ^#^*vs*. PBMSC therapy alone, ^Δ^*vs*. transplantation of *oe*β-catenin-treated MSCs, ^†^*vs*. transplantation of PBMSCs transfected with *oe*β-catenin plus *sh*Oct4 or *sh*β-catenin alone (*n* = 5 per group).

Angiogenesis is a major determinant of myocardial repair induced by MSCs^11,22^; therefore, we investigated the roles of β-catenin/Oct4 signaling in regulating angiogenesis of PBMSCs to provide mechanistic insights into myocardial repair. The blood vascular density was markedly higher in the rats receiving the *oe*β-catenin-treated MSCs than in the animals receiving PBS injection or PBMSCs alone; however, deficiency of β-catenin or Oct4 (*sh*β-catenin or *oe*β-catenin+*sh*Oct4) significantly decreased the blood vascular density (Fig. 8a, b). In addition, the decreased blood vascular density induced by *sh*β-catenin was restored by Oct4 overexpression (*sh*β-catenin+*oe*Oct4). This finding indicated that treatment of PBMSCs with β-catenin overexpression before cell transplantation markedly enhanced their angiogenic potential by activating Oct4.

We next investigated the effect of β-catenin upregulation on the vascularization of transplanted PBMSCs. The vascularization of transplanted cells was evaluated using FACS and immunofluorescence in EGFP-positive cells isolated from the PBMSC-treated infarcted hearts. There was a significant increase in the levels of the vascular endothelial cell markers CD31 and factor VIII in the *oe*β-catenin/*oe*Oct4 group compared with the control vector group, and *sh*β-catenin or *sh*Oct4 appeared to significantly decrease the levels of these proteins (Fig. 8c–e). Therefore, β-catenin/Oct4 is mainly required to enhance the vessel differentiation of PBMSCs in ischemic hearts.

Furthermore, the ELISA results of the supernatant of heart tissues and plasma of rats showed that the effects of β-catenin/Oct4 on the enhancement of PBMSC angiogenesis were reflected in the promotion of their paracrine secretion, as evidenced by increased levels of the proangiogenic cytokines Ang1, bFGF, HGF, and VEGF in the rats that received the *oe*β-catenin/*oe*Oct4-treated MSCs (Fig. 8f–k). In contrast, depletion of Oct4 significantly attenuated the aforementioned secretion by PBMSCs in ischemic hearts and the plasma of rats. Together, these findings imply that β-catenin improves the angiogenesis of PBMSCs by improving their paracrine activity through Oct4 signaling.

## Discussion

MSCs are a strong regenerative material and a potent therapeutic source. However, delivery of MSCs into an ischemic environment provides limited efficacy because of poor survival and low regeneration at the injury site. Therefore, new strategies to improve MSC survival and angiogenesis are needed. A minimally invasive and easy procedure to collect MSCs from peripheral blood would greatly facilitate regenerative and therapeutic outcomes^23,24^. Thus, we collected PBMSCs, which had similar properties to mesenchymal stem cells, from the peripheral blood of rats. Genetically modified PBMSCs overexpressing β-catenin showed enhanced long-term survival and vascular differentiation by activating Oct4-mediated antiapoptotic effects and angiogenesis. Upregulation of β-catenin is a promising strategy to improve the long-term engraftment and myocardial repair of PBMSCs in ischemic hearts. Oct4, as a downstream regulated target of β-catenin, strongly promoted the cytoprotection and paracrine activity of PBMSCs.

In this study, we proposed peripheral blood as an alternative, noninvasive source of rat MSCs on the basis of the following observations. First, PBMSCs were successfully isolated from peripheral arterial blood, which was consistent with our previous study in which MSCs were collected from the peripheral vein and the culprit coronary artery of MI patients^11^. The isolation and culture conditions for PBMSCs have also been described in other studies using peripheral blood as a source^25–27^; thus, we concluded that this tissue is a feasible and appropriate source of MSCs. Second, although MSCs lack unique cell membrane surface markers^28^ and the precise combination of markers is affected by the isolation and cultivation method^29^, in this study, PBMSCs were positive for MSC-associated cell surface markers (CD44, CD90, CD73, and CD105) and showed low expression of HSC markers (CD34 and CD45) and EPC markers (CD133 and CD31), which was consistent with that described for rat bone marrow MSCs^30,31^. The results showed that these cells mainly consisted of MSCs, excluding HSC and EPC contamination of the primary PBMSCs. Third, regarding vascular differentiation, PBMSCs produce vascularization, which is a critical feature of angiogenesis in the ischemic myocardium^32^. The identification of factors that enhance cardiac repair by the restoration of the vascular network is important^33^. Therefore, we concluded that PBMSCs have potential as grafted cells for myocardial repair.

Autologous applications for regenerative therapies are an attractive approach because PBMSCs provide an autologous, effective, alternative, and readily available cell source to treat patients with MI^34^. However, a potential drawback of using PBMSCs is poor survival, which could limit their efficacy for ischemic myocardial repair^35^. In our in vitro experiments, PBMSCs revealed gradual senescence and apoptosis over culture time. Therefore, we developed a novel strategy to promote their long-term survival.

β-catenin, the prime effector of canonical Wnt signaling, expresses numerous genes responsible for cell proliferation, differentiation, and survival^36^. Recent studies have suggested that Wnt/β-catenin signaling maintains the self-renewal and tumorigenicity of head and neck CSCs by activating Oct4^37^. In this study, PBMSCs overexpressing β-catenin showed increased viability/survival and decreased senescence/apoptosis even after 70 days of culturing. These findings were in line with observations in other stem cells^38–40^. Hoffmann et al. found that agonism of Wnt/β-catenin signaling promotes MSC expansion^41^. Moreover, β-catenin deficiency caused the opposite effect, and simultaneous overexpression and knockdown of β-catenin resulted in no significant change. All these data confirm that β-catenin overexpression has long-term advantages for stem cells and can be considered a feasible alternative to improve the viability/survival of PBMSCs in vivo.

Based on the antiapoptotic effect of β-catenin, activation of β-catenin expression showed a more dramatic response to β-catenin overexpression than no intervention (vehicle). β-Catenin signaling maintains the growth of CSCs and directly modulates the expression of Nanog and Oct4^42^. We found that following β-catenin activation in PBMSCs, only Oct4 was produced at high levels. Other stem cell factors showed no significant change in response to altered β-catenin expression, suggesting that β-catenin specifically activates Oct4 expression. Notably, whether the Oct4 levels were high or low, β-catenin levels did not change following alteration of Oct4 expression. However, modification of β-catenin/Oct4 expression caused significant changes in Oct4 expression. The ChIP assay showed that β-catenin directly activated *Oct4* transcription in PBMSCs. These findings provide evidence that Oct4 functions as a downstream effector of β-catenin signaling in PBMSCs. In addition, Oct4 was revealed to function as a downstream target of β-catenin/T-cell factor (TCF) in Wnt-activated mouse embryonic stem cells^43^.

As a transcription factor, Oct4 activates the expression of several downstream genes involved in cell proliferation and survival^11,44^. In the present study, Oct4 overexpression resulted in increased expression levels of the antiapoptotic proteins Bcl2 and survivin and decreased expression levels of the apoptosis-related proteins Bax and caspase-3. Moreover, high expression of Oct4 increased proliferation and decreased apoptosis of PBMSCs. The higher levels of Oct4, Bcl2, and survivin after β-catenin overexpression would explain the long-term engraftment of PBMSCs in the ischemic hearts. In addition, Oct4 overexpression ameliorated the reduced engraftment and apoptosis of PBMSCs induced by β-catenin deficiency, similar to the long-term survival role for Oct4 in embryonic stem cells^45^. Taken together, these findings provide mechanistic insights into the promotive effect of β-catenin/Oct4 signaling on PBMSC long-term survival, thereby illustrating the possibility of activating β-catenin/Oct4 signaling to improve the poor survival of PBMSCs for myocardial repair after MI.

Therefore, we further investigated the effects of β-catenin/Oct4 signaling on myocardial repair induced by PBMSC therapy. The hearts of people with MI undergo cardiac remodeling, characterized by an expanded myocardial infarct size and a dilated left ventricle^46^. A similar finding in the rats with MI receiving PBS injection revealed a progressive decline in cardiac ejection fraction, progressive enlargement of the left ventricle, gradual thinning of the ventricular wall, and expansion of the infarct size. The infarcted hearts clearly showed inflammation and fibrosis. Although transplantation of PBMSCs alone transiently prevented ischemia-induced aggravation of cardiac function and structure at 30 d post-MI, these benefits were not sustained at 90 d post-MI. PBMSC therapy alone did not significantly reduce the infarct size, MI-induced fibrosis, myocyte loss, or inflammation. However, using a lentivirus encoding β-catenin, we demonstrated that β-catenin, via Oct4 signaling, persistently maintained cardiac function and ameliorated cardiac remodeling by improving PBMSC engraftment and decreasing PBMSC apoptosis, which resulted in increased 90-day survival of the rats with MI receiving β-catenin/Oct4-treated MSCs compared with that of the PBS or vehicle control groups. Correspondingly, high β-catenin/Oct4 expression increased the levels of antiapoptotic proteins, supporting the view that β-catenin/Oct4 signaling is associated with long-term engraftment and myocardial repair of PBMSCs in infarcted hearts. Importantly, we showed that *Oct4* knockdown by transfection of an *Oct4* shRNA completely eliminated the cardiac effects of both β-catenin overexpression and PBMSC therapy (*oe*β-catenin+*sh*Oct4), as shown by worsening cardiac function, augmented fibrosis, myocardial infarct size and loss, and decreased myocardial engraftment of PBMSCs. Stimulating β-catenin also helped to sustain somatic cell survival and proliferation by activating Oct4^47^. Collectively, these findings indicated that β-catenin plays a crucial role in rescuing stem cell fate and maintaining cardiac function by inducing an Oct4-dependent antiapoptotic mechanism.

In addition, the β-catenin pathway controls the maintenance of CSCs in many tissues and organs by regulating angiogenesis, epithelial-mesenchymal transition, and the microenvironment^48^. Our results in ischemic hearts revealed that high β-catenin expression resulted in high levels of proangiogenic cytokines and stimulated PBMSC vascular differentiation, which contributed to the formation of blood vessels, indicating the contribution of mesenchymal-endothelial transition (MEndoT) to angiogenesis^49^. In the rats receiving *oe*β-catenin-treated PBMSCs, Oct4 was upregulated in ischemic cardiomyocytes to an optimal extent, which promoted the secretion of proangiogenic cytokines (Ang1, bFGF, HGF, and VEGF). This phenomenon increased angiogenesis, improved cardiac function, and inhibited cardiac remodeling. Furthermore, Oct4 overexpression alleviated *β-catenin* knockdown-induced injury in PBMSCs, as shown by preserved paracrine function and MEndoT. In contrast, Oct4 deficiency completely eliminated PBMSC angiogenesis induced by β-catenin overexpression, as evidenced by decreased vascularization, reduced vascular density, and decreased β-catenin-mediated proangiogenic cytokines. Our previous study demonstrated that Oct4-dependent FoxC1 activation improves the survival and neovascularization of MSCs under myocardial ischemia^50^. All these data might reveal a novel mechanism: β-catenin ameliorated ischemia-related impairment of PBMSC-mediated angiogenesis by inducing MEndoT via an Oct4-dependent mechanism. This issue will be an important subject for future investigation. Our findings provide a rationale for the ongoing clinical evaluation of combinatorial therapies comprising genetic modification and stem cell transplantation^51^.

Two limitations exist in the present study. First, we observed that β-catenin agonists or inhibitors mainly affected β-catenin/Oct4-specific antiapoptotic/proapoptotic protein expression but did not significantly change GSK-3β expression. These findings are inconsistent with those of previous studies. A GSK inhibitor or N-cadherin antibody treatment successfully enhanced β-catenin signaling and resulted in significant increases in mature cardiomyocyte proliferation^52^. Yan et al. demonstrated that N-cadherin overexpression enhances adipose tissue-derived MSC (ADSC)-protective effects against ischemic heart failure via β-catenin-mediated MMP-10/MMP-13/HGF expression and production, promoting ADSC/cardiomyocyte adhesion and ADSC retention^53^. These conflicting reports suggest that the combination of *β-catenin* overexpression with other strategies (e.g., alteration of GSK signaling) may have better benefits for cell therapy and warrants further dedicated investigation. Second, compared with those from uninjured rats, PBMSCs from the peripheral blood of the traumatized rats showed greater proliferation, but they grew slowly and underwent an ~2-fold increase in PD on the 2nd-day post-culture. The average population doubling time of MSCs from adipose, placenta, bone marrow, umbilical cord, and amnion was less than 2 days under appropriate culture conditions^28^. This phenomenon might greatly hinder their clinical application, given that cultures must be expanded to obtain a sufficient number of cells for transplantation. However, cell doubling rapidly increased, by over 2-fold of PD, in less than 2 days after transfection of β-catenin or Oct4. This finding suggests that in vitro culture in combination with genetic engineering can be expanded to generate a sufficient number of PBMSCs for clinical application.

Finally, we showed that β-catenin can improve the long-term survival and angiogenesis of PBMSCs via Oct4 signaling, which considerably promoted the effects of stem cell therapy in ischemic hearts, opening up further options for therapeutic rescue experiments.

## Supplementary information

Supplemental material

## Data Availability

The datasets used and/or analyzed during the current study are available from the corresponding author on reasonable request.

## References

[1] Hayashita-Kinoh H, Takeda S, Okada T (2021). Enhanced cell survival and therapeutic benefits of IL-10-expressing multipotent mesenchymal stromal cells for muscular dystrophy. Stem Cell Res. Ther..

[2] Yin J (2020). ARS2/MAGL signaling in glioblastoma stem cells promotes self-renewal and M2-like polarization of tumor-associated macrophages. Nat. Commun..

[3] Wagne RT, Xu X, Yi F, Merril BJ, Cooney AJ (2010). Canonical Wnt/β-catenin regulation of liver receptor homolog-1 mediates pluripotency gene expression. Stem Cells.

[4] Das PK, Islam F, Lam AK (2020). The roles of cancer stem cells and therapy resistance in colorectal carcinoma. Cells.

[5] Grinat J (2020). The epigenetic regulator Mll1 is required for Wnt-driven intestinal tumorigenesis and cancer stemness. Nat. Commun..

[6] Evangelisti C (2020). Targeting Wnt/β-catenin and PI3K/Akt/mTOR pathways in T-cell acute lymphoblastic leukemia. J. Cell Physio..

[7] Liu HW (2018). The disruption of the β-catenin/TCF-1/STAT3 signaling axis by 4-acetylantroquinonol B inhibits the tumorigenesis and cancer stem-cell-like properties of glioblastoma cells, in vitro and in vivo. Cancers.

[8] Nager M (2012). β-Catenin signalling in glioblastoma multiforme and glioma-initiating cells. Chemother. Res. Pract..

[9] Pádua D, Figueira P, Ribeiro I, Almeida R, Mesquita P (2020). The relevance of transcription factors in gastric and colorectal cancer stem cells identification and eradication. Front. Cell. Dev. Biol..

[10] Zhao R (2014). A nontranscriptional role for Oct4 in the regulation of mitotic entry. Proc. Natl Acad. Sci. USA.

[11] Zhang S (2017). HIF-2α and Oct4 have synergistic effects on survival and myocardial repair of very small embryonic-like mesenchymal stem cells in infarcted hearts. Cell. Death. Dis..

[12] Fazeli Z (2021). Correlation of TCF4, GSK, TERT and TERC expressions with proliferation potential of early and late culture of human peripheral blood mesenchymal stem cells. Cell. J..

[13] Calloni R (2005). Differential expression profiling of membrane proteins by quantitative proteomics in a human mesenchymal stem cell line undergoing osteoblast differentiation. Stem Cells.

[14] Kaiser S (2007). BM cells giving rise to MSC in culture have a heterogeneous CD34 and CD45 phenotype. Cytotherapy.

[15] Nakamura T (2007). Significance and therapeutic potential of endothelial progenitor cell transplantation in a cirrhotic liver rat model. Gastroenterology.

[16] Mihaila SM (2013). Human adipose tissue-derived SSEA-4 subpopulation multi-differentiation potential towards the endothelial and osteogenic lineages. Tissue Eng. Part. A..

[17] Hu X (2015). Dextran-coated fluorapatite crystals doped with Yb3^+^/Ho3^+^ for labeling and tracking chondrogenic differentiation of bone marrow mesenchymal stem cells in vitro and in vivo. Biomaterials.

[18] Martella E (2014). Secreted adiponectin as a marker to evaluate in vitro the adipogenic differentiation of human mesenchymal stromal cells. Cytotherapy.

[19] Livak KJ, Schmittgen TD (2001). Analysis of relative gene expression data using real-time quantitative PCR and the 2(-Delta Delta C(T)) Method. Methods.

[20] Zhang S (2012). Comparison of various niches for endothelial progenitor cell therapy on ischemic myocardial repair: coexistence of host collateralization and Akt-mediated angiogenesis produces a superior microenvironment. Arterioscler. Thromb. Vasc. Biol..

[21] Cao C (2018). Bidirectional juxtacrine ephrinB2/Ephs signaling promotes angiogenesis of ECs and maintains self-renewal of MSCs. Biomaterials.

[22] Xie DM (2021). Cardiac derived CD51-positive mesenchymal stem cells enhance the cardiac repair through SCF-mediated angiogenesis in mice with myocardial infarction. Front. Cell. Dev. Biol..

[23] De Schauwer C (2014). Characterization and profiling of immunomodulatory genes of equine mesenchymal stromal cells from non-invasive sources. Stem. Cell. Res. Ther..

[24] Venugopal B, Mohan S, Kumary TV, Anil Kumar PR (2020). Peripheral blood as a source of stem cells for regenerative medicine: emphasis towards corneal epithelial reconstruction—an in vitro study. Tissue Eng. Regen. Med..

[25] Yan M (2019). Hypoxia reduces the osteogenic differentiation of peripheral blood mesenchymal stem cells by upregulating Notch-1 expression. Connect. Tissue Res..

[26] Liu K (2018). Human peripheral blood-derived mesenchymal stem cells with NTRK1 over-expression enhance repairing capability in a rat model of Parkinson's disease. Cytotechnology.

[27] Du GQ (2021). Concentration changes of peripheral blood mesenchymal stem cells of sprague dawley rats during distraction osteogenesis. Orthop. Surg..

[28] Zhan XS (2019). A comparative study of biological characteristics and transcriptome profiles of mesenchymal stem cells from different canine tissues. Int. J. Mol. Sci..

[29] Havens AM (2013). Human very small embryonic-like cells generate skeletal structures, in vivo. Stem. Cells Dev..

[30] Rose RA (2008). Bone marrow-derived mesenchymal stromal cells express cardiac-specific markers, retain the stromal phenotype, and do not become functional cardiomyocytes in vitro. Stem Cells.

[31] Corcos J (2011). Bone marrow mesenchymal stromal cell therapy for external urethral sphincter restoration in a rat model of stress urinary incontinence. Neurourol. Urodyn..

[32] Castellan RF (2020). miR-96 and miR-183 differentially regulate neonatal and adult postinfarct neovascularization. JCI insight.

[33] Gladka MM (2021). Cardiomyocytes stimulate angiogenesis after ischemic injury in a ZEB2-dependent manner. Nat. Commun..

[34] Pieper IL (2017). Isolation of mesenchymal stromal cells from peripheral blood of ST elevation myocardial infarction patients. Artif. Organs.

[35] Qadura M, Terenzi DC, Verma S, Al-Omran M, Hess DA (2018). Concise Review: Cell therapy for critical limb ischemia: an integrated review of preclinical and clinical studies. Stem cells.

[36] Wang M (2011). Recent progress in understanding molecular mechanisms of cartilage degeneration during osteoarthritis. Ann. N. Y. Acad. Sci..

[37] Lee SH (2014). Wnt/β-catenin signalling maintains self-renewal and tumourigenicity of head and neck squamous cell carcinoma stem-like cells by activating Oct4. J. Pathol..

[38] Smith MK, Koch PJ, Reynolds SD (2012). Direct and indirect roles for β-catenin in facultative basal progenitor cell differentiation. American journal of physiology. Am. J. Physiol. Lung. Cell. Mol. Physiol..

[39] Templin C (2008). Establishment of immortalized multipotent hematopoietic progenitor cell lines by retroviral-mediated gene transfer of beta-catenin. Exp. Hematol..

[40] Li Z (2019). Moderate activation of Wnt/β-catenin signaling promotes the survival of rat nucleus pulposus cells via regulating apoptosis, autophagy, and senescence. J. Cell. Biochem..

[41] Hoffman MD, Benoit DS (2015). Agonism of Wnt-β-catenin signalling promotes mesenchymal stem cell (MSC) expansion. J. Tissue Eng. Regen. Med..

[42] Zhou Y, Li X, Ye M (2021). Morusin inhibits the growth of human colorectal cancer HCT116‑derived sphere‑forming cells via the inactivation of Akt pathway. Int. J. Mol. Med..

[43] Kelly KF (2011). β-catenin enhances Oct-4 activity and reinforces pluripotency through a TCF-independent mechanism. Cell. Stem Cell..

[44] Han JM, Kim HL, Jung HJ (2021). Ampelopsin inhibits cell proliferation and induces apoptosis in HL60 and K562 leukemia cells by downregulating AKT and NF-κB signaling pathways. Int. J. Mol. Sci..

[45] Li Y (2011). Long-term survival of exogenous embryonic stem cells in adult bone marrow. Cell. Res..

[46] Li X, Zhang S, Wa M, Liu Z, Hu S (2019). MicroRNA-101 protects against cardiac remodeling following myocardial infarction via downregulation of runt-related transcription factor 1. J. Am. Heart Assoc..

[47] Li YQ (2018). Exploring confluence-related signalling to modulate the expression of Oct4—a role in facilitating mouse somatic cell reprogramming?. Curr. Stem. Cell. Res. Ther..

[48] Ram Makena M (2019). Wnt/β-catenin signaling: the culprit in pancreatic carcinogenesis and therapeutic resistance. Int. J. Mol. Sci..

[49] Batlle R (2019). Regulation of tumor angiogenesis and mesenchymal-endothelial transition by p38α through TGF-β and JNK signaling. Nat. Commun..

[50] Ji Z (2021). Oct4-dependent FoxC1 activation improves the survival and neovascularization of mesenchymal stem cells under myocardial ischemia. Stem. Cell. Res. Ther..

[51] Bartolucci J (2017). Safety and efficacy of the intravenous infusion of umbilical cord mesenchymal stem cells in patients with heart failure: a phase 1/2 randomized controlled trial (RIMECARD trial [randomized clinical trial of intravenous infusion umbilical cord mesenchymal stem cells on cardiopathy]). Circ. Res..

[52] Fan Y (2018). Wnt/β-catenin-mediated signaling re-activates proliferation of matured cardiomyocytes. Stem. Cell. Res. Ther..

[53] Yan W (2020). N-Cadherin overexpression mobilizes the protective effects of mesenchymal stromal cells against ischemic heart injury through a β-catenin-dependent manner. Circ. Res..

