# Supplementary material for: β-Catenin promotes long-term survival and angiogenesis of peripheral blood mesenchymal stem cells via the Oct4 signaling pathway
Source: Exp Mol Med. 2022 Sep 1;54(9):1434–49. doi: 10.1038/s12276-022-00839-4 (PMC9535028; doi:10.1038/s12276-022-00839-4)
Supplement: Supplementary file 1 — Supplemental material [file 12276_2022_839_MOESM1_ESM.pdf]

RESEARCH DESIGN AND METHODS

Antibodies and other reagents

Supplementary Table 1 presents the sequences of the primers used in this study. **Supplementary Table S2** lists the details of the antibodies. 4′,6-Diamidino-2-phenylindole (DAPI; catalog 28718-90-3) was purchased from Sigma-Aldrich.

Animals

Inbred Lewis rats were used. The Animal Care and Use Committee of GuangZhou Red Cross Hospital Medical College of Ji-Nan University approved all the animal experiments (**Additional file)**, which were carried out in compliance with the Guide for the Care and Use of Laboratory Animals published by The National Academies Press (http://www.nap.edu/).

Isolation, expansion, and purification of PBMSCs

Rats were randomly divided into two groups (n=15, each group): one group of rats underwent surgical trauma on their backs, the other group of rats received no surgery. PBMSCs were isolated from rat abdominal aortic blood, and cultured via the adherent culture method, as described previously [1]. Briefly, mononuclear cells (MNCs) were cloned via limited dilution, and expanded in MSC complete medium (Iscove's modified Dulbecco's medium (IMDM) with 20% fetal bovine serum (FBS), 20 ng/mL bFGF,2 mM L-glutamine, penicillin [100 U/ml], and streptomycin [100 μg/ml]). When cultured for about 21 days, the cell confluence reached 80%, and the cells were digested with 0.25% trypsin. The fourth generation cells were used for subsequent experiments, including their purity, viability, characteristics, pluripotency, and gene

transfection.

Phenotypic characterization of PBMSCs

The characteristics of PBMSCs were determined using fluorescence activated cell sorting (FACS).The characteristics of the MSCs were confirmed using antibodies recognizing MSC-associated cell surface markersCD44, SH3 [CD71], CD90, and SH2 [CD105] [2-3] and hematopoietic markers CD34 and CD45 [4]. Endothelial progenitor cell (EPC) molecular markers CD31 and CD133 were also detected [5]. Mouse IgG1, IgG2a, and IgG2b (Becton Dickinson, Mountain View, CA, USA) were used as isotype controls, and marker expression was evaluated using FACS. Thesamples were analyzed using a flow cytometer (FACSCalibur, BD Biosciences, San Jose, CA, USA) using Cell Questanalytic software.

***In vitro* directed differentiation of PBMSCs**

The PBMSCs underwent direct differentiation toward osteogenesis, chondrogenesis, adipogenesis, and angiogenesis by growth factor supplementation and growth on defined matrices. For osteogenesis, the PBMSCs were induced with a osteogenic differentiation medium kit (HUXUB-90021, Cyagen, Soochow, China) and cultured for 21 days; alizarin red staining was performed to evaluate osteogenic products, as described previously [6]. For chondrogenesis, the PBMSCs were induced for 21 days using a chondrogenic differentiation medium kit (HUXUB-90041, Cyagen) for 14 days and evaluated by alcian blue staining for differentiation identification [7]. For adipogenesis, the PBMSCs were cultured for 21 days in an adipogenic differentiation medium kit (HUXUB-90031, Cyagen). The formation of lipid vacuoles was assessed

by Oil Red O staining [8]. For vascular differentiation, growth factors bFGF (5 ng/ml; Invitrogen), and VEGF (20 ng/ml; R&D Systems; Minneapolis, MN, USA) were added. The capillary-like structures were viewed 6 h later. Microscopic fields containing the tube structures that formed on the gel were photographed using fluorescence inverted phase contrast microscopy. Five fields per test condition were examined [9]. Angiogenesis was detected using immunofluorescence with factor VIII and alpha smooth muscle actin (α-SMA) double positive- staining.

β-catenin and Oct4 transfection

Retroviral plasmid vectors, pMXs, expressing β-catenin or Oct4, were transfected with pReceiver-LV233 lentiviral vector (GeneCopoeia (Rockville, MD, USA) into PBMSCs using the Fugene HD reagent, as directed by the manufacturer's instructions. A pSi- LVRU6GP vector with a puromycin resistance cassette (GeneCopoeia) was used to express short hairpin (sh)RNAs to knockdown *β-catenin* (encoding β-catenin) or *Oct4* expression. β-catenin (*oe*β-catenin) or Oct4 (*oe*Oct4) overexpression and β-catenin (*sh*β-catenin) or Oct4 (*sh*Oct4) deficiency were induced by transfecting the cells with vectors encoding *β-catenin* or *Oct4*, *β-catenin* shRNA, or *Oct4* shRNA, respectively, control cells were transfected with control vectors.

Cell grouping settings

To determine the effect of β-catenin on the proliferation and survival of PBMSCs, the cells were randomly divided into the following four groups according to different intervention methods: The vehicle group (control group), the β-catenin overexpression group (*oe*β-catenin), the *β-catenin* knockdown group (*sh*β-catenin), and the β-catenin

overexpression+*β-catenin* knockdown group (*oe*β-catenin plus *sh*β-catenin). To determine the effect of Oct4 on the proliferation and survival of PBMSCs, the cells were divided into: The vehicle group, the Oct4 overexpression group (*oe*Oct4), the *Oct4* knockdown group (*sh*Oct4), and the Oct4 overexpression+*Oct4* knockdown group (*oe*Oct4 plus *sh*Oct4). To evaluate the intermodulation effects of *β- catenin* and *Oct4* in cell growth and apoptosis, we added *oe*β- catenin+*sh*Oct4 and *sh*β- catenin+*oe*Oct4. After transfection, all groups of PBMSCs were cultured for 70 days for subsequent experiments to analyze the growth and apoptosis of PBMSCs.

To investigate the mechanisms of β-catenin/Oct4-mediated cytoprotective effects against apoptotic cell death, indirection activation of the Wnt/β-catenin pathway was carried out by adding 40μM β-catenin agonist (SKL2001; 40μM; MedChemexpress, Monmouth Junction, NJ, USA) or β-catenin inhibitor (FH535; 5 μM; MedChemexpress), followed by observation of the expression of anti-apoptotic and proapoptotic molecules. Then, we performed gain- of-function experiments to directly activate the β-catenin/Oct4 signaling pathway by transfecting the cells with vectors encoding β-catenin/Oct4 (*oe*β-catenin/*oe*Oct4), and loss-of-function experiments by lentiviruses carrying targeted *β-catenin*/*Oct4*shRNA (*sh*β-catenin/*sh*Oct4) .

MTS assay

For the cell viability assay, PBMSCs were seeded at 2 × 10^3 cells/well on a 96-well plate at 37 °C and cultured for 48 h. Cell viability was determined by the 3-(4,5-dimethylthiazol-2-yl)-5-(3-carboxymethoxyphenyl)-2-(4-sulfophenyl)-2H-tetra zolium (MTS) method in accordance with the CellTiter 96 Aqueous One Solution

Viability assay manual (Promega Corporation, Madison, WI, USA).

Growth curve of PBMSCs

PBMSCs were seeded at a density of 2 × 10^3/well onto 24-well plates at 5% CO_2 and 37 °C. The PBMSCs were digested and counted in each group at each time point accurately (1, 2, 3, 4, 5, 6, and 7 d). According to the number of cells corresponding to each time point, the growth curve was drawn and the population doubling of PBMSCs was determined.

Colony forming assay

For each group, 500 cells were transferred to 6 well-plates after transfection. After 7 days of culture, the medium was removed, the cells were washed in two PBS washes and fixed/stained with 4% paraformaldehyde (Sigma, St. Louis, MO, USA) and 0.5% crystal violet (Sigma) solutions for 30 min at room temperature. The plates were then rinsed with PBS and left to dry overnight. A cell mass containing more than 50 cells was considered a cell colony; the number of cell colonies was counted under a phase contrast microscope (CH20CH30CK2, Olympus, Tokyo, Japan).

Senescence-associated β-galactosidase activity

1 × 10^5/well PBMSCs were seeded in 6 well-plates. When the cells were cultured for 2 days, the cells were washed twice with PBS and fixed with 4% paraformaldehyde. Cells on slides were sealed with parafilm and stained with X-gal staining solution (Cell Signal Technology) for 24 h in an incubator with no CO_2 at 37 °C. The stained and unstained cells were counted under a phase contrast microscope (Olympus). The percentage of senescent cells (blue) to the total cell number in the field of view was

calculated.

Apoptosis detected by flow cytometry.

Cells subjected to cell cycle analysis were trypsinized, fixed with 70% ethanol at 4 °C overnight, and stained with Annexin V-APC and propidium iodide (PI) in PBS at 37 °C for 30 min. Cell populations were analyzed by flow cytometry using a flow cytometer (FACSCalibur, BD Biosciences). 10000 cells were counted in each sample. Data were analyzed using BD ModFit LT version 3.3 (BD Biosciences).

Chromatin immunoprecipitation assays (CHIP)

About 2.0×10^6 cells were used in each ChIP experiment. The proteins were cross- linked by incubating cells with 1% formaldehyde for 10 min. Nuclear extraction and chromatin digestion was then performed. Sheared chromatin was diluted and immunoprecipitated with 2 μg of an anti-β-catenin or control IgG antibody. DNA- protein complexes were then eluted and purified. ChIP assays were performed according to manufacturer's protocol from the ChIP assay kit (Merck Millipore,Billerica, MA, USA). The DNA samples were detected by using real-time PCR analysis. To amplify the β-catenin binding site in the *Oct4* promoter, the primers sequences shown in **Supplementary Table 1** were used.

Immunofluorescence staining.

Cells or tissues seeded or fixed on lysine-treated glass coverslips were washed with PBS, fixed with 4% paraformaldehyde (Sigma), permeabilized by 0.5% Triton X-100 for 10 min, and pre-blocked with 5% Bovine serum albumin for 1 h.

Immunofluorescence staining was performed by incubation with antibodies recognizing β-catenin, Oct4, Factor VIII, α-SMA, Angiopoietin 1 (Ang1), or VEGF (**Supplementary Table 2**) at 4 °C overnight, followed incubation with secondary antibodies: Alexa-488 anti-mouse (A32723; 1:200; Thermo Fisher Scientific, Inc., Waltham, MA, USA.) or Alexa-594 anti-rabbit (A32732; 1:200; Thermo Fisher Scientific, Inc.) for 1 h at room temperature. The cells were washed extensively with 0.1% tween-20 in PBS and mounted with 4',6-Diamidino-2-phenylindole dihydrochloride (DAPI, S33025; Thermo Fisher Scientific, Inc.). Fluorescent signals were captured by an Olympus FV1000 confocal microscope (Olympus) and analyzed using FV10-ASW software (Olympus).

Quantitative Reverse-transcription PCR (qRT-PCR)

Total RNA was extracted from cells or tissues using the TRIzol reagent (Thermo Fisher Scientific, Inc.) and converted to cDNA using PrimeScript RT Master Mix (Takara Bio, Inc., Dalian, China). qPCR with the cDNA as the template was performed using SYBR Premix ExTaq (Takara Bio, Inc.) using the qTOWER version 3.0 PCR system (Jena Industries, Inc., Ontario, Canada). The thermocycling conditions were as follows: 95 °C for 2 min for pre-denaturation; then 40 cycles of denaturation at 95°C for 34 sec, and annealing and extension at 55 °C for 5 sec. The forward and reverse primers for all the target genes are presented in **Supplementary Table 1**. The expression of all genes was calculated using the $2^{-\Delta\Delta Ct}$ method using *Gapdh* as the control [10].

Western blotting analysis

Cells or tissues were collected for protein extraction and analysis. Total protein was extracted using Radioimmunoprecipitation assay (RIPA) cell lysis buffer (P0013C, Beyotime, Shanghai, China). The protein concentration of the cell or tissue lysate was examined using a bicinchoninic acid (BCA) protein assay (Pierce Biotechnology, Rockford, IL, USA). 30 μg of protein was denatured, resolved in a 12% SDS-PAGE minigel, and electrophoretically transferred onto polyvinylidene fluoride (PVDF) membranes (Millipore). The membranes were pre-blocked with 5% nonfat dry milk for 1 h and hybridized with antibodies (**Supplementary Table 2**) against β-catenin, Oct4, bFGF, survivin, Bcl2, Bax, cleaved-caspase-3, GSK-3α/β and phospho-GSK-3α/β at 4 °C overnight. The membranes were washed with PBS containing 0.1% Tween-20, and then incubated with diluted horseradish peroxidase (HRP)-labeled goat anti-rabbit IgG solutions (ARG65351; 1:3000; Arigo Biolaboratories, Hsinchu City, Taiwan) at room temperature for 1 h. GAPDH was used as the loading control. The immunoreactive protein bands were revealed using an ECL Western Blotting Substrate (Thermo Fisher Scientific, Inc.) kit and quantified using Image lab system version 2.0 (Bio-Rad Laboratories, Inc., Hercules, CA, USA).

EGFP labelling

At 24 h after transfection with the *oe*β-catenin, *sh*β-catenin, *oe*Oct4, *sh*Oct4, or control vectors, cells were co-transfected with a lentiviral vector containing enhanced GFP (EGFP) cDNA, as described previously [11]. More than 70% of MSCs were EGFP- positive, as determined by flow cytometry.

Rat MI model, cell therapy, and groups

Inbred Lewis rats were used. After anesthesia with intraperitoneal injection of 3% sodium pentobarbital, MI was induced in the rats by ligating the left anterior descending coronary artery. Animals with an ejection fraction (EF) < 70% and fractional shortening (FS) < 35%, evaluated by echocardiography after induction of MI, were selected. After establishment of the MI model, the animals randomly received injection of PBS, or PBMSCs pre-treated with *oe*β-catenin, *sh*β-catenin, *oe*Oct4, *sh*Oct4, or control vectors. Cell transplantation was achieved by injection into the infarct and peri-infarct regions (5×10^6 cells, four sites, two sites per infarct or peri- infarct area, 20 ml per site, 1-2 cm apart). To minimize postoperative pain, 2.5% bupivacaine was sprayed at the point of incision immediately before closure, and buprenorphine hydrochloride (0.03 mg/kg) was administered intramuscularly. After the final layer of skin was closed, triple antibiotic ointment (neomycin sulfate, polymyxin B sulfate, and bacitracin zinc) was applied to the wound. Cyclosporin A (Novartis Pharma, Basel, Switzerland) was administered daily (5 mg/kg, i.h.) from the first day after MI until the animals were sacrificed on day 30. Finally, twenty animals were studied in each subgroup.

Echocardiography

Under general anesthesia, as described above, rats underwent echocardiography in a 7.5-MHz phased-array transducer (Acuson Sequoia 256, Siemens,Malvern, PA, USA) operated by an experienced technician blinded to the treatment group identity. Two- dimensional images were obtained at the mid-papillary and apical levels. The left ventricular end-diastolic volume (LVEDV) and internal diameter at diastolic

phase and (LVEDD) were measured using the biplane area-length method. LV FS was calculated according to the modified Simpson method: FS (%) = [(LVIDd-LVIDs)/LVIDd] × 100, where LVID is the LV internal dimension, s is systole, and d is diastole. All measurements were averaged for three consecutive cardiac cycles.

Histology

At the end of each study (90 days after cell transplantation), rats were then sacrificed by cervical dislocation, and then the hearts were perfused with 4% buffered formalin, harvested, and sectioned into 2 transverse slices parallel to the atrioventricular ring. Myocardial tissue sections were randomly chosen from five animals for triphenyltetrazolium chloride (TTC) staining. The infarct size was determined by calculating the percentage of the infarcted area against the whole LV area using ImageJ (NIH, Bethesda, MD, USA). The slides were stained with Masson's Trichrome staining and hematoxylin and eosin (H&E), and by immunofluorescence, photographed, and analyzed using planimetry software (Sigma Scan Pro version 5,Systat Software, Inc., San Jose, CA, USA). The stained sections were used to measure the infarct size, and the average ratio of the scar area to the entire LV cross-sectional area (percent scar/LV area) and the average ratio of the viable myocardium to the LV scar area.In each case, five independent images from each area were analyzed from each section.

Terminal Deoxynucleotidyl Transferase dUTP Nick End Labeling (TUNEL)

The apoptosis of transplanted PBMSCs in the infarcted hearts was detected using a

TUNEL assay according to the standard procedure. After fixing in 4% paraformaldehyde, the tissues were stained by 50 μL TUNEL reaction mixture (Roche, Basel, Switzerland) for 60 min at 37 °C. The cell nuclei were stained using DAPI and observed under an Olympus microscope (Olympus FV1000). The apoptosis rate of transplanted PBMSCs was expressed as the percentage of $TUNEL^{+}EGFP^{+}$cells relative to all $EGFP^{+}$ cells in the ischemic hearts after 90days of transplantation.

Enzyme-linked immunosorbent assays (ELISA)

Plasma samples were obtained before the rats were sacrificed. Plasma samples were obtained before the rats were sacrificed. The levels of pro- angiogenic cytokines Ang1, bFGF, HGF, and VEGF in the plasma of rats and the supernatant of heart tissues were analyzed using an ELISA kit. Assays were conducted in 96-well microplates according to the manufacturer's instructions (GloMax®-Multi+, Promega, USA).

Engraftment and vasculogenesis

Cells were collected from the left ventricles of five randomly selected hearts per experimental group, as previously described [9]. Briefly, the heart was excised and subjected to retrograde perfusion with Ca^{2+}-free perfusion buffer. The left ventricle was minced in collagenase, and the solution was filtered through a nylon mesh. The collected cells were washed with PBS and analyzed using flow cytometry (Becton Dickinson). Engraftment was evaluated by determining the proportion of cells that expressed EGFP relative to all isolated ventricular cells. Heart tissues were immunostained with anti-cardiomyocyte marker troponin T antibodies to verify the retention of transplanted EGFP-prelabeled PBMSCs and counterstained with DAPI to

detect the nuclei. The cells (2×10^5/ml) were then incubated with 4 ml of antibodies against factor VIII. Vascular differentiation was evaluated by calculating the proportion of cells that expressed both EGFP and factor VIII relative to all EGFP-positive cells.

Statistical analysis

Data are presented as the mean ± standard error of the mean (SEM). Discrete variables are presented as the frequency and proportion. After performing a normality test (Shapiro–Wilk test) and homogeneity test of variance, the data that satisfieda normal distribution and equal variance assumptions were used for one-way analysis of variance (ANOVA) of these variables. When the data were conformed to a normal distribution but non-homogeneity of variance, Welch ANOVA was performed. Comparisons were performed using the chi-squared or Fisher's exact test for discrete variables. A 95% confidence interval (CI) ($p < 0.05$) was considered significant.

.

References

1. Fazeli Z. *et al*. Correlation of TCF4, GSK, TERT and TERC Expressions with Proliferation Potential of Early and Late Culture of Human Peripheral Blood Mesenchymal Stem Cells. *Cell. J.* **22,** 431-436 (2021).

2. Calloni, R., Viegas, G.S., Türck, P., Bonatto, D. & Pegas Henriques, J.A. Mesenchymal stromal cells from unconventional model organisms. *Cytotherapy.* **16**, 3-16 (2014).

3. Foster L.J. *et al*. Differential expression profiling of membrane proteins by

quantitative proteomics in a human mesenchymal stem cell line undergoing osteoblast differentiation. *Stem Cells.* **23**,1367-1377 (2005).

4. L Ramos T. *et al*. MSC surface markers (CD44, CD73, and CD90) can identify human MSC-derived extracellular vesicles by conventional flow cytometry. *Cell. Commun. Signal.***14**, 2 (2016).

5. Nakamura T. *et al*. Significance and therapeutic potential of endothelial progenitor cell transplantation in a cirrhotic liver rat model. *Gastroenterology,* **133**, 91-107 (2007).

6. Mihaila S. M. *et al*. Human adipose tissue-derived SSEA-4 subpopulation multi-differentiation potential towards the endothelial and osteogenic lineages. *Tissue. Eng. Part. A.* **19,** 235-246 (2013).

7. Hu X. *et al*. Dextran-coated fluorapatite crystals doped with Yb3+/Ho3+ for labeling and tracking chondrogenic differentiation of bone marrow mesenchymal stem cells in vitro and in vivo. *Biomaterials*. **52,** 441-451 (2015).

8. Martella E. *et al*. Secreted adiponectin as a marker to evaluate in vitro the adipogenic differentiation of human mesenchymal stromal cells. *Cytotherapy.* **16,** 1476-1485 (2014).

9. Zhang S. *et al*. HIF-2α and Oct4 have synergistic effects on survival and myocardial repair of very small embryonic-like mesenchymal stem cells in infarcted hearts. *Cell. Death. Dis.* **8,** e2548 (2017).

10. Livak, K. J. & Schmittgen, T. D. Analysis of relative gene expression data using real-time quantitative PCR and the 2(-Delta Delta C(T)) Method. *Methods.* **25,**

402-408 (2001).

11. Zhang S. *et al.* Comparison of various niches for endothelial progenitor cell therapy on ischemic myocardial repair: coexistence of host collateralization and Akt-mediated angiogenesis produces a superior microenvironment. *Arterioscler. Thromb. Vasc. Biol.* **32,** 910-923 (2012).

Supplementary Figure and Table Legends

Supplementary Fig. 1. β-Catenin specifically activates Oct4 expression. The mRNA and protein expression levels of stem cell-related transcription factors Oct4, c- Myc, Nanog, and KLF4 in PBMSCs receiving vehicle, *oe*β-catenin, *si*β-catenin, or *oe*β-catenin plus *si*β-cateninwere assessed using qRT-PCR (**a**) and western blotting (**b**). The transfection of *oe*β-catenin significantly increased the expression of Oct4, but did not significantly increase the expression of c-Myc, Nanog, and KLF4. Graphical data show the means ± SEM. $P < 0.05$: **vs.*vehicle group, $^{\#}$*vs. oe*β-cateningroup, $^{\Delta}$*vs.sh*β-cateningroup, $n = 10$ in each group. **c** Representative images of β-catenin (red) and Oct4 (green) immunofluorescencestaining. DAPI nuclear staining (blue) is also shown, scale bar = 20 μm. β-catenin expression was detectable in the cytoplasm and nucleus of PBMSCs, while Oct4 was mainly expressed in the nucleus. Note that β-catenin overexpression markedly increased expression of both β-catenin and Oct4, whereas β-catenin knockdown significantly decreased their expression. **Supplementary Fig. 2. The effects of the β-catenin agonist or inhibitor on the β- catenin/Oct4 signaling pathwayin PBMSCs.** The protein levels of β-catenin/Oct4

signaling molecules (β-catenin, Oct4, bFGF, survivin, Bcl2, Bax, caspase-3, GSK3β, and phospho-GSK3 Beta (pGSK3β) in PBMSCs receiving vehicle (CON), the β-catenin agonist, the β-catenin inhibitor, or the β-catenin agonist plus inhibitor, as assessed using western blotting. Representative western blot (**a**) and the subsequent quantification (**b**) showing that the β-catenin agonist significantly increased the levels of β-catenin, Oct4, survivin, and Bcl2, and decreased the levels of Bax and caspase-3. The β-catenin inhibitor had the opposite effect. However, β-catenin agonist or inhibitor did not significantly alter the level of phosphorylated (p)-GSK3 β /GSK3β. The ratio of p-GSK3 β : total GSK3β was calculated. Graphical data show the means ± SEM. $P < 0.05$: **vs.*vehicle group (CON), $^{\#}$*vs.*β-catenin agonist, $^{\Delta}$*vs.*β-catenin inhibitor, n = 10 in each group.

Supplementary Fig. 3. Effect of the direct activation or inactivation of the β-catenin/Oct4 signaling pathway on the growth and apoptosis of PBMSCs under hypoxic culture. Gain or loss-of-function experiments to specifically evaluate the effects on the growth and apoptosis of PBMSCs by transfecting the cells with vectors encoding β-catenin/Oct4 (*oe*β-catenin/*oe*Oct4) or lentivirus carrying targeted β-catenin/Oct4 shRNA (*sh*β-catenin/*sh*Oct4). **a**, PBMSCs were cultured for 7 days under hypoxic conditions after gain or loss-of-function experiments, and the number of cumulative population doublings was determined. **b**, Apoptosis of cardiac cells was assessed by Annexin V/PI staining. The apoptosis level of all groups of PBMSCs was detected using flow cytometrywith Annexin V/ PI staining. **c**, Representative flow cytometry images of cells are shown after 7 days under hypoxic

conditions. All graphical data show the means ± SEM. $P < 0.05$: * *vs.* vehicle group (without transfection (-)), $^{\#}$ *vs.* *oe*β-catenin, *oe*Oct4, or *sh*β-catenin+*oe*Oct4, $^{\Delta}$ *vs.* *sh*β-catenin or *oe*β-catenin +*sh*Oct4, $n = 5$ in each group.

Supplementary Table 1. Primers for qRT-PCR analysis of rat tissues.

Supplementary Table 2. Antibodies for fluorescence activated cell sorting (FACS), western blotting (WB), enzyme linked immunosorbent assay (ELISA), and immunofluorescence (IF).

Supplementary Fig. 1

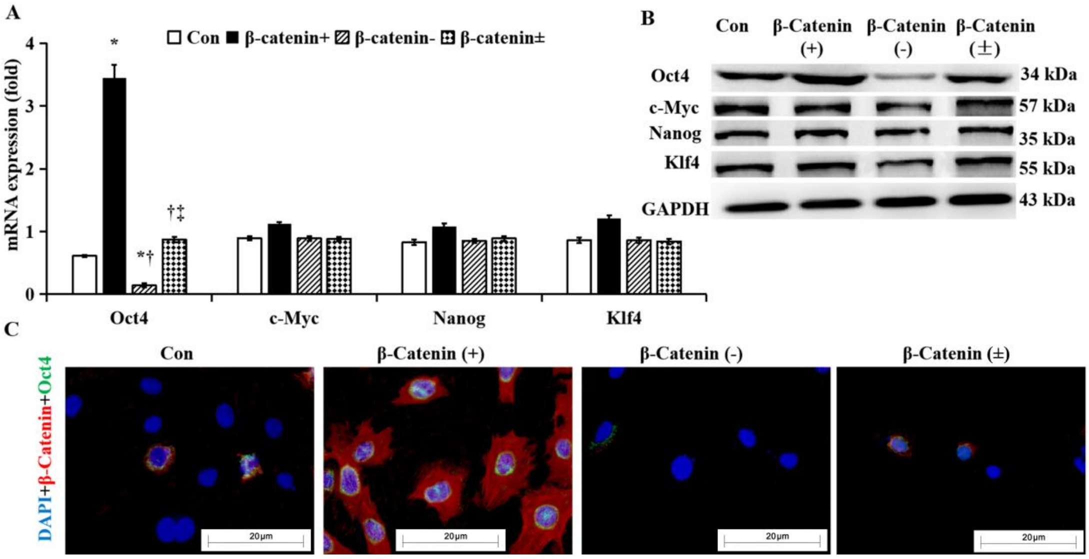

Supplementary Fig. 2

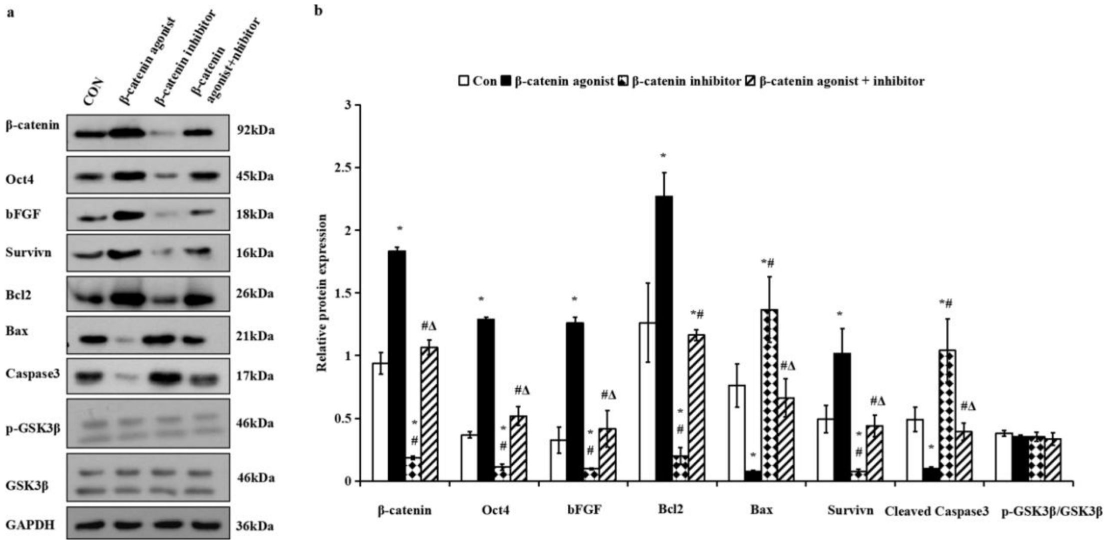

Supplementary Fig. 3

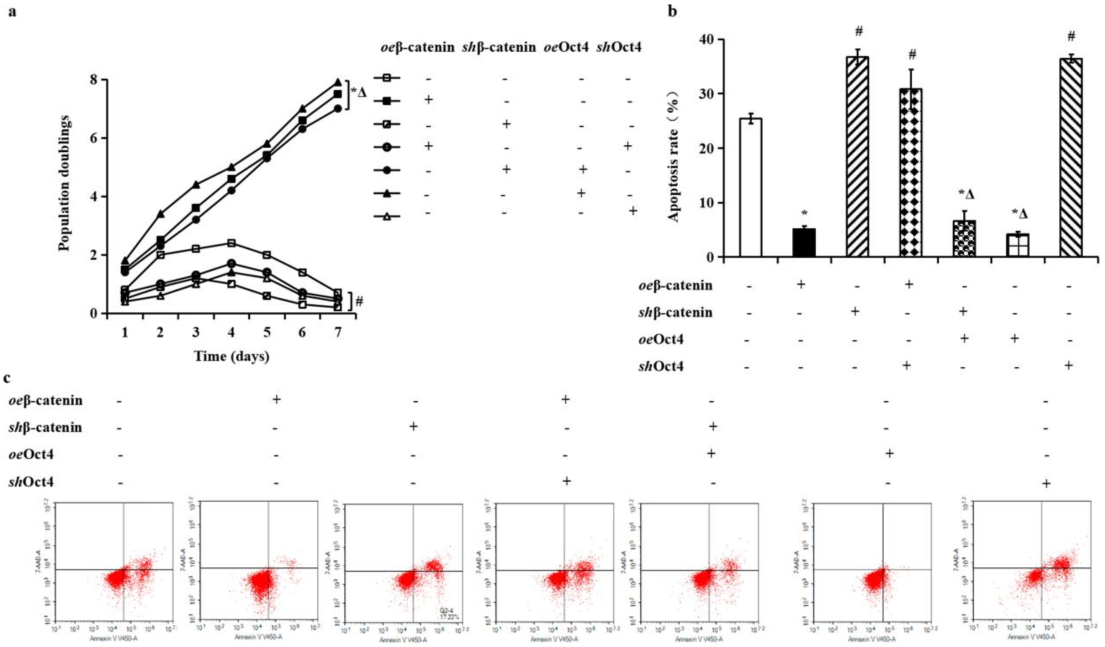

Supplementary Table 1 Sequences of primers used for gene amplification.

Genes	Forward	Reverse
β-catenin	5′-TCAACGGCTCCAGCAAGAACAAG-3′	5′-CTCCGCCTCCTCCACGAAGG-3′
Oct4	5′-CCTTGTCCCACTTGCTGGTT-3′	5′-AGCAAAGACGGGGCAAGAAA-3′
bFGF	5′- AGGCAGGAAGGGAGAAAGTTG-3′	5′- GAATCTGTCCCGTTCGGCG-3′
Survivin	5′- TGCGCCTTCCTTACAGTCAA-3′	5′- CCCACCCATAGATCCTGTCA-3′
Bcl2	5′- TTGATCTCAAGGCGGGGATG-3′	5′- GGTTGGCAAGGCGTGATCTA-3′
Bax	5′-CGGGTGGTTGCCCTTTTCTA -3′	5′-TGTCCAGCCCATGATGGTTC-3′
GAPDH	5′- GCATCTTCTTGTGCAGTGCC-3′	5′- TACGGCCAAATCCGTTCACA-3′

Supplementary Table 2 The antibodies for FACS, WB, ELISA, and IF

Name	Description	Company	Catalogue number	Applications
α-SMA	Alpha-smooth muscle actin	Millipore	MAB1522	WB
Ang1	Angiopoietin 1	Biorbyt<br>RayBiotech	orb10091<br>ELR-Angiopoietin1-1	WB, IF, ELISA
Bax	BCL2-Associated X	Cell Signaling Technology	2772S	WB, IF, ELISA
Bcl2	B-cell lymphoma-2	Cell Signaling Technology	50E3	WB, IF
bFGF	Basic fibroblast growth factor	Cell Signaling Technology<br>RayBiotech	20102<br>ELR-bFGF-1	WB, IF<br>ELISA
Caspase-3	Cleaved-caspase-3	Cell Signaling Technology	9661S	WB, IF
β-Catenin		Cell Signaling Technology	8480	WB, IF, ELISA
CD31	Cluster of differentiation 31	Bioss Inc.	bs-0468R-PE	FCM
CD44	Cluster of differentiation 44	Bioss Inc.	bs-2507R-PE	FCM
CD90	Cluster of differentiation 90	Becton Dickinson	561973	FCM
CD34	Cluster of differentiation 34	Becton Dickinson	751621	FCM
CD45	Cluster of differentiation 45	GeneTex	GTX44565	FCM, IF
CD71	Cluster of differentiation 71	MyBioSource.com	MBS215085	FCM
CD105	Cluster of differentiation 105	Biorbyt	orb488557	FCM
CD133	Cluster of differentiation 133	Biorbyt	orb703845	FCM
c-Myc		Abcam	ab32072	WB, IF
Factor VIII	Von willebrand factor (vWF)	Abcam	ab5694	WB, IF
GAPDH	Glyceraldehyde-3- phosphate dehydrogenase	Cell Signaling Technology	5174S	WB
GSK-3α/β	Glycogen synthase kinase-3α/β	Cell Signaling Technology	5676	WB
Phospho-GSK-3α/β	Phospho-glycogen synthase kinase-3α/β	Cell Signaling Technology	9327	WB
HGF	Hepatocyte growth factor	RayBiotech	ELR-HGF-1	ELISA, WB
Ki-67		Abcam	ab15580	IF
Klf4	Kruppel-like factor 4	Boster	BA3453	WB,IF
Nanog		Novus Biologicals	NBP1-77109	WB, IF
Oct4	Octamer- binding protein 4	Cell Signaling Technology	2750	WB, IF
Survivin		Cell Signaling Technology	2808S	WB, IF
Troponin T		Biorbyt	orb182936-CF488A	IF
VEGF	Vascular endothelial growth factor	Bioss Inc.<br>LifeSpan BioSciences	bs-0279R<br>LS-F542	WB, IF, ELISA

Abbreviations: FACS: fluorescence activated cell sorting; WB: western blot; ELISA: enzyme linked immunosorbent assay; IF: immunofluoroscence.
